# Efficacy and safety of intraoperative hyperthermic intraperitoneal chemotherapy for locally advanced colorectal cancer (HIPECT4): final analysis of randomized clinical trial

**DOI:** 10.1093/bjsopen/zrag002

**Published:** 2026-03-27

**Authors:** Alvaro Arjona-Sánchez, Alberto Gutiérrez-Calvo, Juan J Segura-Sampedro, Rafael Morales, Estibalitz Pérez-Viejo, Vanessa Concepción-Martín, Susana Sánchez-García, Alfonso García-Fadrique, Isabel Prieto-Nieto, Lana Bijelic, Juan Torres-Melero, Maria Ramirez-Faraco, Arancha Prada-Villaverde, Joaquin Carrasco-Campos, Manuel Artiles-Armas, Pedro Villarejo-Campos, Gloria Ortega-Pérez, Enrique Boldo-Roda, Juan M Sánchez-Hidalgo, Angela Casado-Adam, Lidia Rodríguez-Ortiz, Blanca Rufian-Andújar, Enrique Aranda, Maria T Cano-Osuna, Cesar Díaz-López, Antonio Romero-Ruiz, Maria Carmen Vazquez-Borrego, Sebastian Rufián-Peña

**Affiliations:** Unit of Oncologic and Pancreatic Surgery, University Hospital Reina Sofía, Córdoba, Spain; Maimónides Biomedical Research Institute of Córdoba (IMIBIC)/Reina Sofia University Hospital/University of Córdoba, Cordoba, Spain; Unit of Peritoneal Oncologic Surgery, Surgery Department, Hospital Príncipe de Asturias, Alcalá de Henares, Madrid, Spain; General & Digestive Surgery Service, Hospital Universitario Son Espases, Palma de Mallorca, Spain; School of Medicine, Universidad CEU San Pablo, 28003 Madrid, Spain; General & Digestive Surgery Service, Hospital Universitario La Paz, Madrid, Spain; Unit of Oncologic and Pancreatic Surgery, Hospital Son Spaces, Palma de Mallorca, Spain; Unit of Oncologic Surgery, Hospital University Fuenlabrada, Madrid, Spain; Unit of Peritoneal Oncologic Surgery and Colorectal Surgery, Hospital University Nuestra Señora de la Candelaria, Tenerife, Spain; Unit of Surgery, Hospital University Ciudad Real, Ciudad Real, Spain; Department of Surgery, Instituto Valenciano de Oncología, Valencia, Spain; Unit of Oncologic Surgery, University Hospital La Paz, Madrid, Spain; Unit of Surgery, Consorci Sanitari Integral, Hospital de Sant Joan Despí Moises Broggi, Barcelona, Spain; Unit of Surgery, Hospital de Torrecárdenas, Almería, Spain; Unit of Oncologic Surgery, University General Hospital Reina Sofia, Murcia, Spain; Unit of Surgery, Hospital University Infanta Cristina, Badajoz, Spain; Unit of Surgery, Hospital Regional University of Malaga, Malaga, Spain; Department of General and Digestive Surgery, University Hospital Gran Canaria Dr. Negrín, Las Palmas de Gran Canaria, Canary Islands, Spain; Unit of Oncologic Surgery, Hospital Fundación Jimenez Diaz, Madrid, Spain; Unit of Surgery, MD Anderson Cancer Center, Madrid, Spain; Unit of Surgery, Hospital Provincial Castellón, Castellón, Spain; Unit of Oncologic and Pancreatic Surgery, University Hospital Reina Sofía, Córdoba, Spain; Maimónides Biomedical Research Institute of Córdoba (IMIBIC)/Reina Sofia University Hospital/University of Córdoba, Cordoba, Spain; Unit of Oncologic and Pancreatic Surgery, University Hospital Reina Sofía, Córdoba, Spain; Maimónides Biomedical Research Institute of Córdoba (IMIBIC)/Reina Sofia University Hospital/University of Córdoba, Cordoba, Spain; Unit of Oncologic and Pancreatic Surgery, University Hospital Reina Sofía, Córdoba, Spain; Maimónides Biomedical Research Institute of Córdoba (IMIBIC)/Reina Sofia University Hospital/University of Córdoba, Cordoba, Spain; Unit of Oncologic and Pancreatic Surgery, University Hospital Reina Sofía, Córdoba, Spain; Maimónides Biomedical Research Institute of Córdoba (IMIBIC)/Reina Sofia University Hospital/University of Córdoba, Cordoba, Spain; Maimónides Biomedical Research Institute of Córdoba (IMIBIC)/Reina Sofia University Hospital/University of Córdoba, Cordoba, Spain; Unit of Medical Oncology, University Hospital Reina Sofia, Córdoba, Spain; Maimónides Biomedical Research Institute of Córdoba (IMIBIC)/Reina Sofia University Hospital/University of Córdoba, Cordoba, Spain; Unit of Medical Oncology, University Hospital Reina Sofia, Córdoba, Spain; Unit of Oncologic and Pancreatic Surgery, University Hospital Reina Sofía, Córdoba, Spain; Maimónides Biomedical Research Institute of Córdoba (IMIBIC)/Reina Sofia University Hospital/University of Córdoba, Cordoba, Spain; Maimónides Biomedical Research Institute of Córdoba (IMIBIC)/Reina Sofia University Hospital/University of Córdoba, Cordoba, Spain; Unit of Oncologic and Pancreatic Surgery, University Hospital Reina Sofía, Córdoba, Spain

## Abstract

**Background:**

Despite adjuvant systemic chemotherapy after surgical resection in patients with pT4 stage colon cancer, a high percentage of them will develop peritoneal metastases. Intraoperative hyperthermic intraperitoneal chemotherapy (HIPEC) is a treatment option with the goal of preventing metachronous peritoneal metastases. The aim of this study was to report the longer-term outcomes of peritoneal control with the use of intraoperative HIPEC based on mitomycin C after the last enrolled patient of the HIPECT4 trial reached 36 months follow-up.

**Methods:**

Between November 2015 and March 2021, patients with resectable primary clinical T4 N0-2M0 were included and randomized (1:1) to either adjuvant HIPEC with mitomycin C (30 mg/m^2^, 60 minutes) or standard treatment. The primary endpoint was locoregional control at 36 months. Kaplan–Meier survival analysis with a log-rank test was used to compare the two study groups.

**Results:**

A total of 184 patients were included and followed up at 36 months. The locoregional control rate was improved with HIPEC compared with adjuvant chemotherapy alone (hazard ratio 0.19, 95% confidence interval 0.04 to 0.86; *P* = 0.031). Three years overall survival and disease-free survival did not differ between patients assigned to the HIPEC and control groups. Subgroup analysis showed better locoregional control with the use of HIPEC for patients with definitive pT4 colon cancer (hazard ratio 0.08, 0.01 to 0.65; *P* = 0.017) and per protocol (receiving adjuvant chemotherapy) patients (hazard ratio 0.18, 0.04 to 0.83; *P* = 0.028). The pattern of recurrence was modified significantly using HIPEC with less peritoneal relapse.

**Conclusion:**

The long-term outcome analysis of the HIPECT4 trial demonstrated that using mitomycin C-based HIPEC reduced peritoneal recurrence in patients with locally advanced colon cancer without increasing toxicity. However, there was no difference in overall survival and disease-free survival.

**Registration number:**

NCT02614534 (https://clinicaltrials.gov/).

## Teaser

Hyperthermic intraperitoneal chemotherapy in prophylaxis reduces locoregional relapse in locally advanced colon cancer, demonstrated in results from long-term follow-up.

## Introduction

Colorectal cancer is a highly prevalent disease, with an estimated incidence of 44 000 new cases in Spain in 2024^1^. The peritoneum is a common site of dissemination^2^, with significantly shorter survival rates than other isolated metastatic sites^3^. Many strategies are aimed at preventing or detecting peritoneal metastases at an early stage. Identifying patients at high risk of developing peritoneal recurrence is crucial to achieving an appropriate therapeutic strategy. Locally advanced colon cancer (pT4) is recognized as an independent risk factor for peritoneal recurrence (local or disseminated)^4^, with a reported incidence of up to 36% at 3 years^2,4^.

Despite adjuvant systemic chemotherapy after surgical resection in patients with pT4 stage colon cancer, a high percentage of them will develop peritoneal metastases. The use of intraoperative hyperthermic intraperitoneal chemotherapy (HIPEC) to prevent metachronous peritoneal metastases has been evaluated in two randomized trials, COLOPEC^5,6^ and HIPECT4^7^, with opposing results. The COLOPEC trial^6^ found no difference in peritoneal metastasis-free survival when oxaliplatin-based HIPEC was used. However, the HIPECT4 trial^7^ showed a clinical benefit in reducing peritoneal recurrence when mitomycin C-based HIPEC was used after surgical resection compared with the standard arm in early reported results. The use of HIPEC to prevent metachronous peritoneal recurrence remains controversial. An important point may be the selection of patients who will benefit more from this proactive approach, as it was shown that patients with right-sided and pT4 colon cancer had the highest benefit in a recent individual patient meta-analysis^8^ that combined both trials.

The aim of this study was to determine peritoneal control with the use of intraoperative HIPEC based on mitomycin C after the last enrolled patient of the HIPECT4 trial reached 36 months follow-up. This final analysis adds more robust evidence to the previous published preliminary results from the HIPECT4 trial^7^ regarding the effect of HIPEC with mitomycin C on locoregional control in patients with locally advanced colorectal cancer. A subgroup analysis was also performed.

## Methods

The study design describing the inclusion and exclusion criteria of the HIPECT4 trial has been published previously^7,9^. Briefly, between November 2015 and March 2021, patients with resectable primary clinical T4 N0-2M0 were included and randomized (1:1) to either adjuvant HIPEC with mitomycin C (30 mg/m^2^, 60 minutes) with standard adjuvant chemotherapy or standard adjuvant chemotherapy alone. The primary endpoint was locoregional control at 36 months, defined as the absence of peritoneal tumour recurrence at 36 months. Disease-free survival (DFS) and overall survival (OS) were secondary endpoints. Morbidity and toxicity have been previously evaluated (Protocol in Suppl_3)^7^.

Median follow-up was estimating using the reverse Kaplan–Meier method. Kaplan–Meier survival analysis with a log-rank test was used to compare the two study groups. All hypothesis contrasts were two-sided. *P* < 0.05 was considered statistically significant. The proportional hazards assumption was tested using Schoenfeld residuals. Additionally, the restricted mean survival time (RMST) and permutation test^10^ were calculated at 12, 24, and 36 months. The number of permutations was 10^4^. The method selected was to average the RMSTs derived from methods 2 and 3. Method 2 extends the survival curve to tau, and method 3 switches the last censored observation to the event observation. RMST avoids the proportionality issues related to the Cox model^11^ (Suppl_1).

Sensitivity analyses were performed. The variables perforation, well/poorly differentiated, and primary tumour location were included in the analysis as covariates. Hazard ratios (HRs) are presented with 95% confidence intervals (c.i.). Additionally, analysis was performed by subgroups (pT4 and per protocol populations (adjuvant treatment received)). Analysis was performed using R software (R Foundation for Statistical Computing, Vienna, Austria. https://www.R-project.org/) (Suppl_1).

## Results

**Fig. 1:**
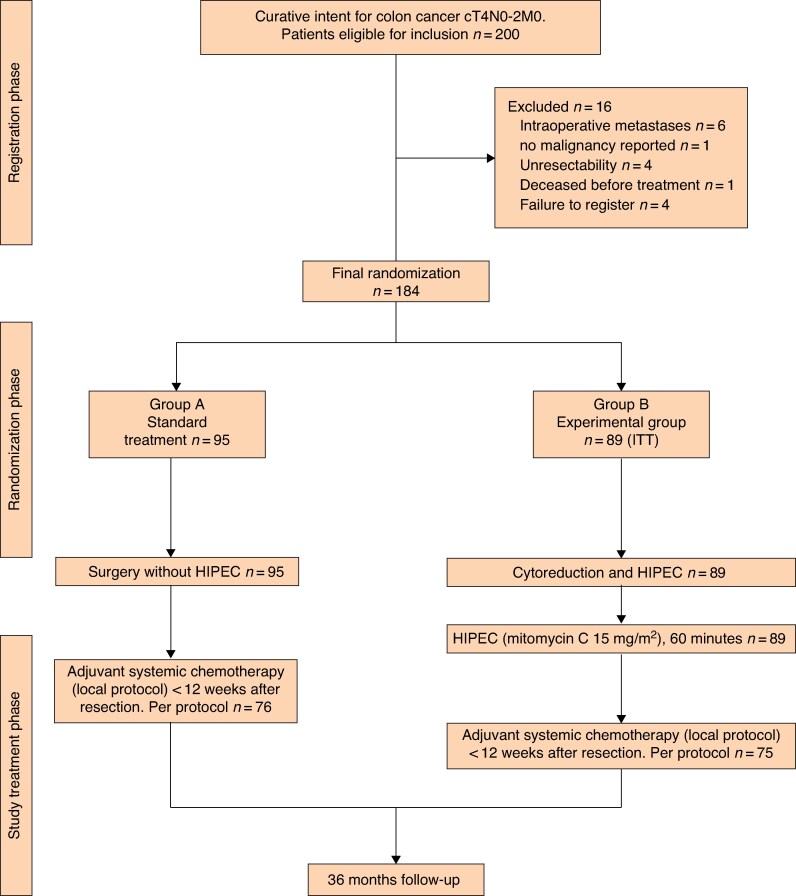
CONSORT diagram ITT, intention-to-treat; HIPEC, hyperthermic intraperitoneal chemotherapy.

In total, 89 patients were assigned to the experimental arm (HIPEC followed by adjuvant chemotherapy) and 95 to adjuvant chemotherapy alone (*Fig. 1*). *Table 1* shows the characteristics of the 184 included patients. In total, 96% of patients were followed for up to 36 months.

**Table 1:** Characteristics of patients included in HIPECT4 trial

	Total (*n* = 184)	HIPEC group (*n* = 89)	Comparator group (*n* = 95)
Age (years), mean(s.d.)	61.5(9.2)	60(8.7)	62(10.6)
**Sex**			
Female	73 (39.7%)	33 (37.1%)	40 (42.1%)
Male	111 (60.3%)	56 (62.9%)	55 (47.9%)
**ECOG performance status**			
0	127 (69.9%)	63 (70.8%)	64 (67.4%)
1	49 (26.6%)	23 (25.8)	26 (27.4%)
2	8 (4.3%)	3 (3.4%)	5 (5.3%)
BMI ≥ 30 kg/m^2^	41 (22.3%)	20 (22.5%)	21 (22.1%)
ASA score ≥ 3	56 (30.4%)	28 (31.4%)	28 (30.1%)
PSS ≥ 1	40 (21.7%)	19 (21.3%)	21 (22.1%)
**Tumour location**			
Right colon	70 (38.0%)	35 (39.2%)	35 (36.8%)
Transverse colon	8 (4.3%)	3 (3.4%)	5 (5.3%)
Left colon	31 (16.9%)	17 (19.1%)	14 (14.7%)
Sigmoid rectum	75 (40.8%)	34 (38.2%)	41 (43.2%)
Preoperative CEA (ng/ml), mean(s.d.)	17.7(49.2)	13(41.2)	22(56.5)
Preoperative Ca 19.9 (UI/ml), mean(s.d.)	26(56.2)	21(37.9)	30(69.3)
**pT category**			
pT1–2	5 (2.7%)	0 (0.0%)	5 (5.4%)
pT3a	18 (9.8%)	9 (10.1%)	9 (9.6%)
pT3b	35 (19.1%)	17 (19.1%)	18 (19.1%)
pT4a	77 (42.1%)	36 (40.4%)	41 (42.1%)
pT4b	48 (26.2%)	27 (30.3%)	21 (22.3%)
**pN category**			
pN0	97 (53.3%)	45 (51.1%)	52 (55.3%)
pN1	43 (23.6%)	23 (26.1%)	20 (21.3%)
pN2	42 (23.1%)	20 (22.7%)	22 (23.4%)
Low-grade differentiation	119 (66.9%)	58 (65.9%)	61 (67.8%)
**Type of adenocarcinoma**			
Mucinous	32 (17.5%)	15 (16.9%)	17 (18.1%)
Signet ring	3 (1.6%)	2 (2.2%)	1 (1.1%)
Tumour perforation	31 (16.8%)	14 (15.7%)	17 (17.9%)
Pathology lymphatic invasion	80 (43.7%)	41 (46.1%)	39 (41.5%)
Pathology perineural invasion	77 (42.3%)	36 (40.9%)	41 (43.6%)
Pathology vascular invasion	66 (36.3%)	31 (34.8%)	35 (37.6%)
Microsatellite instability	34 (18.9%)	22 (25%)	12 (13%)
Tumour size (cm), mean(s.d.)	6.9(3.8)	6.5(3.2)	7.2(4.4)
*BRAF* mutation*, mean(s.d.)	8(22.8)	4(23.5)	4(22.0)
*K-RAS* wild type†, mean(s.d.)	26(59.0)	11(50.0)	15(75.0)
Number of lymph nodes isolated, mean(s.d.)	25(12.6)	25(12.8)	26(12.5)
Blood needed	23 (12.4%)	15 (16.9%)	8 (8.3%)
Laparoscopic approach	29 (15.8%)	17 (19.1%)	12 (12.6%)
Operative time (min), mean(s.d.)	250(97.3)	311(81.8)	193(73.2)
Ostomy	12 (6.5%)	4 (4.4%)	8 (8.4%)
Intraoperative vasoactive drugs	10 (5.4%)	7 (7.9%)	3 (3.1%)
Adjuvant therapy (FOLFOX/CAPOX) Number of cycles, mean(s.d.)	128 (69.6%)	63 (70.7%) 7.2(3.0)	65 (68.4%) 6.9(3.4)

Values are *n* (%) unless otherwise stated. HIPEC, hyperthermic intraperitoneal chemotherapy, s.d., standard deviation; ECOG, Eastern Cooperative Oncologic Group; BMI, body mass index; ASA, American Society of Anesthesiologists; PSS, previous surgical score; CEA, carcinoembryonic antigen; min, minutes. *Calculated on 37 patients studied. †Calculated on 44 patients studied. Comparative analysis using χ^2^ test; *P* < 0.05.

**Fig. 2:**
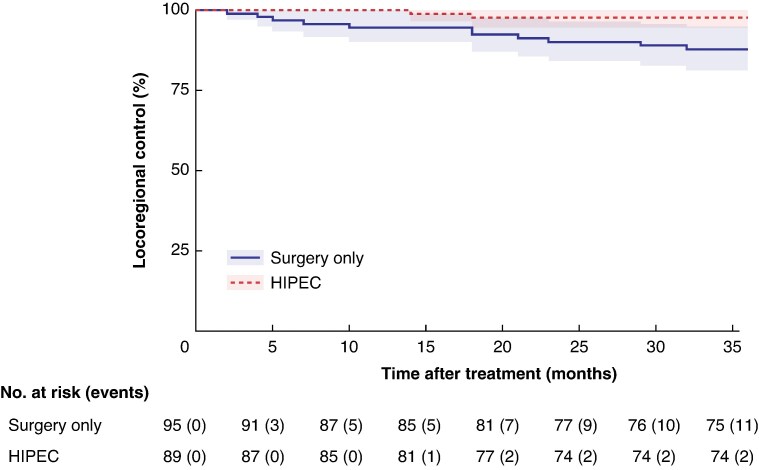
Kaplan–Meier survival curves comparing locoregional control between different HIPEC treatment groups The x-axis represents time after treatment in months, and the y-axis represents the probability of locoregional control. The plot includes confidence intervals and censor marks, with the risk table displaying the number of patients at risk and cumulative events at each time point. Hazard ratio 0.19 (95% confidence interval 0.04 to 0.86); *P* = 0.031. HIPEC, hyperthermic intraperitoneal chemotherapy.

The locoregional control rate was improved with HIPEC compared with adjuvant chemotherapy alone (HR 0.19, 95% c.i. 0.04 to 0.86; *P* = 0.031). Goodness of fit was established using the Schoenfeld residuals test (*P* = 0.887). The RMST (95% c.i.) was calculated at 12, 24, and 36 months in the control group obtaining 11.65 (11.33 to 11.98), 22.82 (21.95 to 23.7), and 33.5 (31.96 to 35.05) months, respectively. The RMST (95% c.i.) at 12, 24, and 36 months in the HIPEC group was 12 (12 to 12), 23.8 (23.53 to 24.08), and 35.51 (34.83 to 36.18) months, respectively. The differences in RMST between groups (HIPEC−control) at these time points were 0.35 (*P* = 0.039), 0.98 (*P* = 0.041) and 2.01 (*P* = 0.020) months, respectively (*Fig. 2*) (*Suppl_S1*).

**Fig. 3:**
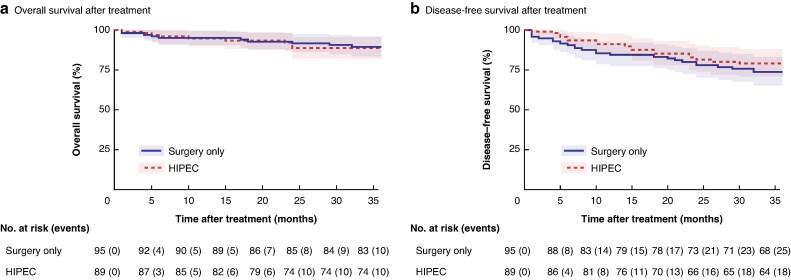
Kaplan–Meier survival curves comparing OS and DFS between different HIPEC treatment groups The x-axis represents time after treatment in months, and the y-axis represents the probability of **a** OS and **b** DFS. The plot includes confidence intervals and censor marks, with the risk table displaying the number of patients at risk and cumulative events at each time point. OS, overall survival; DFS, disease-free survival; HIPEC, hyperthermic intraperitoneal chemotherapy.

Three-year OS and DFS did not differ between patients assigned in the HIPEC and control groups. The OS and DFS RMSTs (95% c.i.) at 36 months were 33.64 (32.08 to 35.20) and 29.96 (27.67 to 32.26) months, respectively, in the control group and 33.45 (31.84 to 35.06) and 31.42 (29.38 to 33.45) months, respectively, in the HIPEC group; the differences were not significant (*P* = 0.86 and *P* = 0.35, respectively) (*Fig. 3*) (*Suppl_S1*).

**Fig. 4:**
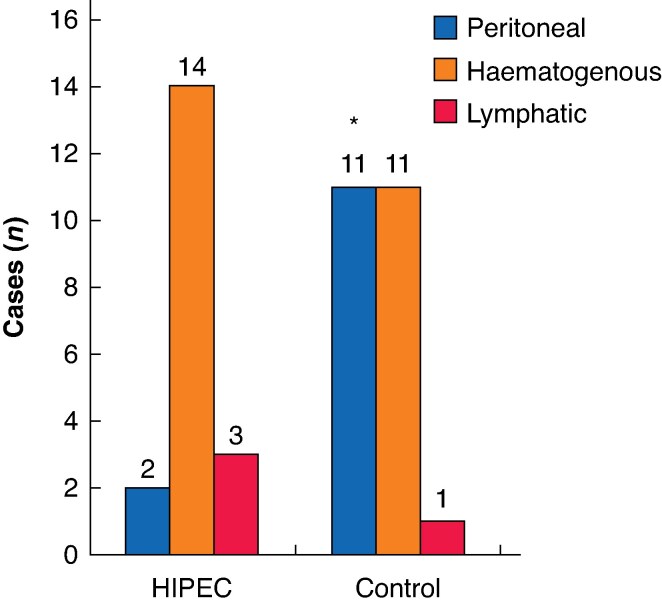
Pattern of recurrence from the HIPECT4 trial Subtypes of haematogenous relapses included in the analysis (control/HIPEC): liver (8/11), lung (2/2), and brain (1/1). HIPEC, hyperthermic intraperitoneal chemotherapy. *Fisher’s exact test: *P* = 0.02.

Subgroup analysis showed better locoregional control with the use of HIPEC for definitive pT4 colon cancer (HR 0.08; 95% c.i. 0.01 to 0.65; *P* = 0.017) and per protocol (receiving adjuvant chemotherapy) patients (HR 0.18, 0.04 to 0.83; *P* = 0.028). DFS did not reach statistical differences in these subgroups with the use of HIPEC; however, a trend in favour of HIPEC was shown in both subgroups: adjuvant therapy (HR 0.53, 0.26 to 1.06; *P* = 0.072) and pT4 stage (HR 0.54, 0.27 to 1.07; *P* = 0.079) (Suppl_1). The pattern of recurrence was modified from peritoneal as predominant in the control group to a systemic pattern in the HIPEC group (*P* = 0.02) (*Fig. 4*).

## Discussion

This final analysis of the HIPECT4 trial confirms the preliminary analysis of the primary endpoint and shows a benefit of HIPEC in locoregional control at 36 months compared with standard of care (surgery + adjuvant chemotherapy alone) without increasing the morbidity or toxicity. However, OS and DFS at 36 months did not reach statistical significance between groups. Patients with T4 colon cancers are at known risk of developing peritoneal carcinomatosis. This type of metastasis has a worse prognosis compared with haematogenous or lymphatic metastases. HIPEC with mitomycin C has demonstrated a change in the pattern to less peritoneal and more systemic relapse^3^. These findings support the use of HIPEC with mitomycin C to prevent peritoneal recurrence in locally advanced colon cancer.

This is the first positive trial demonstrating the benefit of HIPEC based on mitomycin C in colon cancer, with no toxicity for its use in this prophylactic context^7^. Although several groups have adopted this strategy to manage cT4 colon cancer, real-world experience is still needed. However, the use of HIPEC with a short course of oxaliplatin has failed to demonstrate a benefit in different trials evaluating HIPEC in different therapeutic roles: one as adjuvant therapy in peritoneal carcinomatosis^6^ and the other as prophylaxis^5,12^.

The management of locally advanced colon cancer has not changed for decades, using surgery and adjuvant chemotherapy based on FOLFOX (oxaliplatin, fluorouracil, and folinic acid) or CAPOX (capecitabine and oxaliplatin)^13^. Adjuvant chemotherapy in high-risk stage II and stage III colon cancer has demonstrated benefit including mucinous cancers^14^. However, new strategies, mainly peritoneal, are needed to reduce the risk of recurrence for these patients. Neoadjuvant chemotherapy or HIPEC are the main options for management. The use of neoadjuvant chemotherapy has been demonstrated to reduce the recurrence in locally advanced colorectal cancer in the FOXTROT trial^15^ using FOLFOX 4 (HR 0.72, 95% c.i. 0.54 to 0.98; *P* = 0.037). However, other studies such as the OPTICAL trial^16^ did not find better DFS with the use of FOLFOX 6 before surgery, even though the pattern of relapse did not reduce peritoneal recurrence. The use of targeted therapy using monoclonal antibodies such as panitumumab in *RAS/RAF* wild types has also not demonstrated any benefit in locally advanced colorectal cancer in the neoadjuvant setting^17^. New approaches must be investigated for T4 colon cancer. Combination and bidirectional therapy could improve locoregional and systemic control using both neoadjuvant and adjuvant chemotherapy with HIPEC^18^. FOXHIPECT4^18^ is an ongoing phase III trial that combines neoadjuvant chemotherapy using FOLFOX 6 and HIPEC with mitomycin C. The main endpoint is DFS with experimental arms including one with HIPEC and one without, the hypothesis being that the pattern will demonstrate less peritoneal relapse in the HIPEC arm.

One limitation to adopting this strategy as standard clinical practice is the lack of benefit in DFS or OS in this trial. As in the COLOPEC trial^5^, HIPECT4^7^ selected a primary endpoint based on the locoregional effect of HIPEC. This surrogate endpoint can assess the ability of HIPEC to prevent peritoneal recurrence. Considering that peritoneal recurrence has an important negative impact on survival, this surrogate endpoint might be related to survival^3^. The evaluation of a locoregional effect could provide relevant information to reduce the time and the number of patients included in this trial, which are two important aspects for future trials.

The main limitation with the use of simultaneous HIPEC in this prophylactic context would be the classification of patients with cT4 colon cancer based on radiological findings. In the HIPECT4 trial^7^, the accuracy was 69% at accurately predicting pT4, with most of the overstaged patients being classified as pT3 (29%). The FOXTROT trial^15^ evaluated the use of neoadjuvant therapy in locally advanced colon cancer, demonstrating accurate radiological staging of 76% including high-risk pT3 and pT4 (for HIPECT4, predictive positive value for pT3/pT4 was 98%), and the study used a team-trained radiologist. The accuracy to detect T3 or T4 was high, with a positive predictive value of 94.5%; individual T or N was not so identified. The authors recommend using T3–T4 as the indication for neoadjuvant therapy^19^. This would suggest a significant number of patients could be overtreated in the prophylactic setting and the importance of evaluating the risk/benefit for patients within the multidisciplinary team.

Patient selection is crucial for personalized treatment. These include tumoral factors, microsatellite stability, and *RAS/RAF* mutations that influence the effectiveness of different treatments. Patients with right and T4 colon cancer are in a subgroup associated with better locoregional control with the use of HIPEC^8^. A metanalysis from the COLOPEC and HIPECT4 studies demonstrated a benefit for this subgroup of patients. The mismatch repair status has become an essential factor for the use of immunotherapy, potentially avoiding the need for surgery in patients that have a complete clinical response^20^. These factors must be considered for the selection of treatments and future study designs.

This mature outcome analysis of the HIPECT4 trial demonstrates the benefit of using mitomycin C-based HIPEC to prevent peritoneal recurrence in patients with locally advanced colon cancer without increasing toxicity. However, more diagnostic accuracy is needed to identify which patients could benefit more from this therapeutic strategy, incorporating factors other than imaging such as omic analysis in genes, proteins, or radiological images.

## Supplementary Material

zrag002_Supplementary_Data

## Data Availability

Data available on request from corresponding author. Access data are deidentified participant data. The protocol is attached as *Supplementary material*. Researchers may access the data after proposed scientific use of the data has been approved, with investigator support, with a signed data access agreement.
