# Supplementary material for: Efficacy and safety of intraoperative hyperthermic intraperitoneal chemotherapy for locally advanced colorectal cancer (HIPECT4): final analysis of randomized clinical trial
Source: BJS Open. 2026 Mar 27;10(2):zrag002. doi: 10.1093/bjsopen/zrag002 (PMC13023033; doi:10.1093/bjsopen/zrag002)
Supplement: zrag002_Supplementary_Data [file zrag002_supplementary_data.zip › Consort Flow Chart.docx]

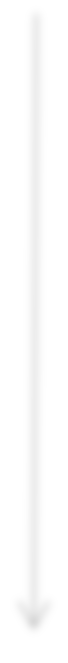

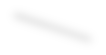

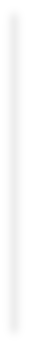

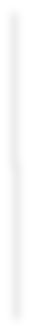

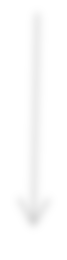

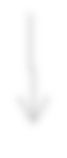

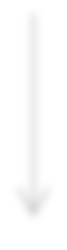

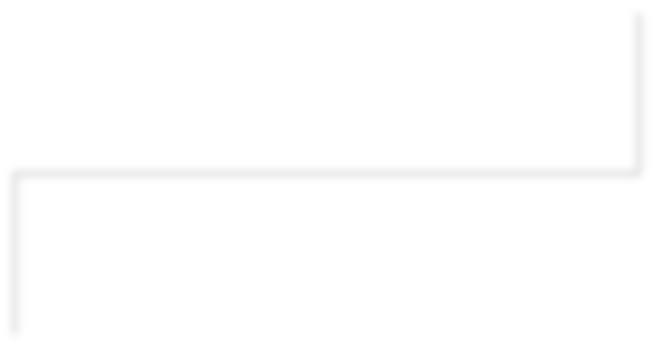

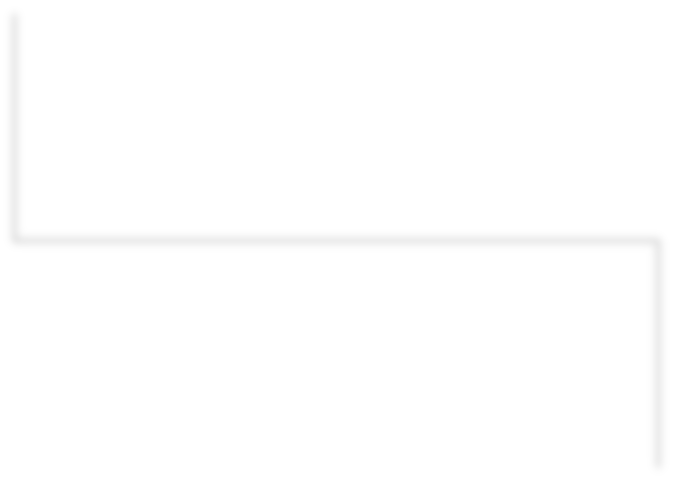

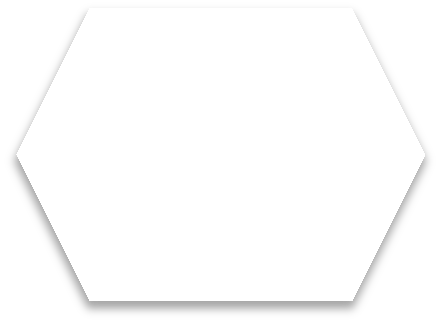

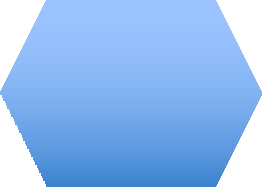

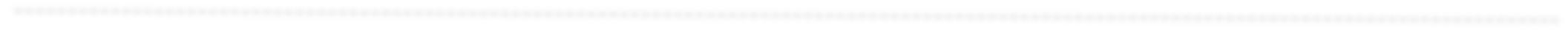

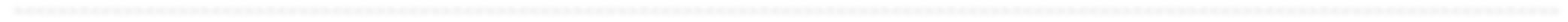

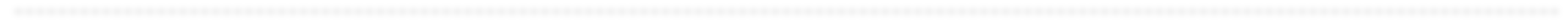


Study treatment phase

Randomisation phase


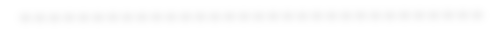


**Curative intent for colon cancer cT4N0-2M0: Eligible for inclusion: 200 patients**

**Excluded patients: 16**

Registration phase

**Intraoperative metastases: 6 No malignity reported: 1 Unresectablity: 4**

**Deceased before treatment: 1 Failure to registration: 4**

Final Randomisation N= 184

**Group A:** Standard treatment N= 95 (ITT)

**Group B:** Experimental group N=89 (ITT)

Surgery: 95 patients

Cytoreduction and HIPEC (89)

HIPEC ( Mitomycin C 15 mg/m2), 60 minutes (89)

Adjuvant systemic chemotherapy (local protocol) < 12 weeks after resection. ( 76 per protocol)

Adjuvant systemic chemotherapy (local protocol),

< 12 weeks after resection (75 pe rprotocol)

36 months follow-up
