# Supplementary material for: Efficacy and safety of intraoperative hyperthermic intraperitoneal chemotherapy for locally advanced colorectal cancer (HIPECT4): final analysis of randomized clinical trial
Source: BJS Open. 2026 Mar 27;10(2):zrag002. doi: 10.1093/bjsopen/zrag002 (PMC13023033; doi:10.1093/bjsopen/zrag002)
Supplement: zrag002_Supplementary_Data [file zrag002_supplementary_data.zip › Supplementary_material.docx]

#### Title: Efficacy and safety of Intraoperative Hyperthermic Intraperitoneal Chemotherapy (HIPEC) for Locally Advanced Colorectal Cancer (HIPECT4): Final analysis of a randomized trial.

*Authors:* A Arjona-Sánchez* PhD^1,2^, A Gutiérrez-Calvo PhD^3^, R. Morales PhD^4^, E Pérez-Viejo PhD^5^, V Concepción-Martín MD^6^, S Sánchez-García PhD^7^, A García-Fadrique PhD^8^, I Prieto-Nieto PhD^9^, L Bijelic PhD^10^, J Torres-Melero PhD^11^, M Ramirez-Faraco MD^12^, A Prada-Villaverde MD^13^, J Carrasco-Campos MD^14^, M Artiles-Armas MD^15^, P Villarejo-Campos PhD^16^, G Ortega-Pérez PhD^17^, E Boldo-Roda MD^18^, JM Sánchez-Hidalgo PhD^1,2^, A Casado-Adam PhD^1,2^, L Rodríguez-Ortiz MD^1,2^, E Aranda PhD^2,19^, MT Cano-Osuna MD^2,19^, C Díaz-López PhD^1^, A Romero-Ruiz PhD^2^, MC Vazquez-Borrego PhD^2^, S Rufián-Peña PhD^1^; on behalf of Spanish Group of Peritoneal Oncologic Surgery (GECOP).

**Affiliations:**

1.- Unit of Oncologic and Pancreatic Surgery, University Hospital Reina Sofía, Córdoba, Spain

2.- Maimónides Biomedical Research Institute of Córdoba (IMIBIC)/Reina Sofia University Hospital/ University of Córdoba, Spain.

3.- Unit of Peritoneal Oncologic Surgery, Surgery department, Hospital Príncipe de Asturias, Alcalá de Henares, Madrid, Spain.

4.- Unit of Oncologic and Pancreatic Surgery, Hospital Son Spaces, Palma de Mallorca, Spain.

5.- Unit of Oncologic Surgery, Hospital University Fuenlabrada, Madrid, Spain.

6.- Unit of Peritoneal Oncologic Surgery and Colorectal Surgery, Hospital University Nuestra Señora de la Candelaria, Tenerife, Spain.

7.- Unit of Surgery, Hospital University Ciudad Real, Ciudad Real, Spain.

8.- Department of Surgery, Instituto Valenciano de Oncología, Valencia, Spain.

9.- Unit of Oncologic Surgery, University Hospital La Paz, Madrid, Spain.

10.- Unit of Surgery, Consorci Sanitari Integral, Hospital de Sant Joan Despí Moises Broggi,

Barcelona,Spain.

11.- Unit of Surgery, Hospital de Torrecárdenas, Almería, Spain.

12.- Unit of Oncologic Surgery, University General Hospital Reina Sofia, Murcia, Spain.

13.- Unit of Surgery, Hospital University Infanta Cristina, Badajoz, Spain.

14.- Unit of Surgery, Hospital Regional University of Malaga, Spain.

15.- Department of General and Digestive Surgery, University Hospital Gran Canaria Dr. Negrín, Las Palmas de Gran Canaria, Canary Islands, Spain.

16.- Unit of Oncologic Surgery, Hospital Fundación Jimenez Diaz, Madrid, Spain.

17.- MD Anderson Cancer Center, Madrid, Spain.

18.- Unit of Surgery, Hospital Provincial Castellón, Castellón, Spain.

19.- Unit of Medical Oncology, University Hospital Reina Sofia, Córdoba, Spain.

**Word count:** 1242.

**Corresponding author:** Alvaro Arjona-Sánchez, MD, PhD Unit of Oncologic and Pancreatic Surgery, H.U. Reina Sofia, Córdoba, Spain e-mail: [alvaroarjona@hotmail.com](mailto:alvaroarjona@hotmail.com),Address: Menendez Pidal Av. 14004, Córdoba, Spain

**Supplementary Materials – Index**

**Supplementary Flow Chart CONSORT Pag 3.**

**Supplementary Methods and Results. Statistical analysis by external service Pag 4**

**Supplementary Trial Protocol HIPECT4 Pag 13**

**Suppplementary CONSORT RCT checklist Pag 69**


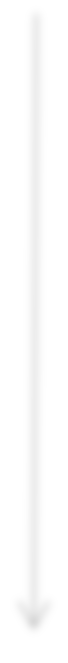

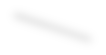

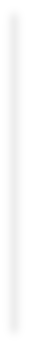

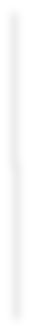

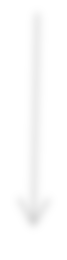

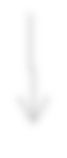

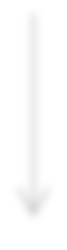

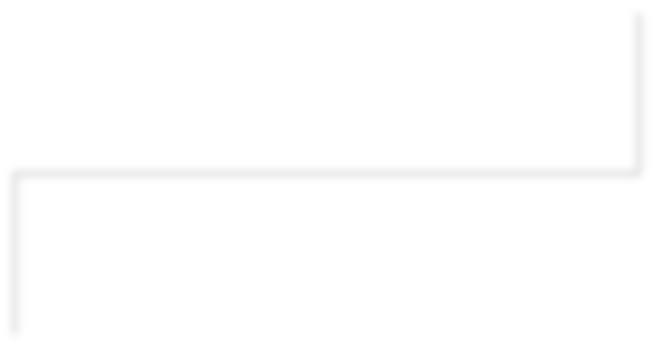

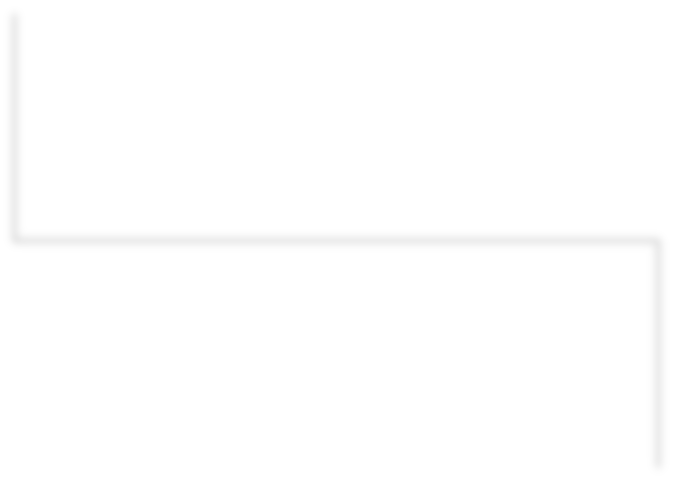

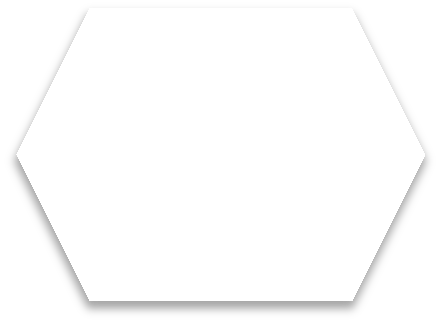

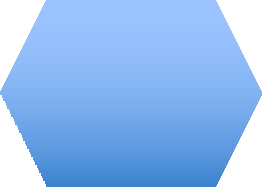

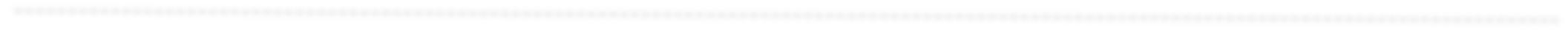

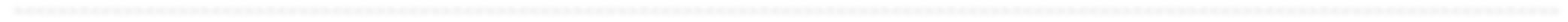

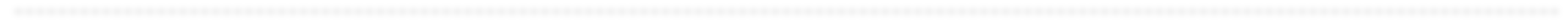


Study treatment phase

Randomisation phase


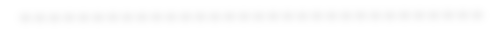


**Curative intent for colon cancer cT4N0-2M0: Ellegible for inclusion: 200 patients**

**Excluded patients: 16**

Registration phase

**Intraoperative metastases: 6 No malignity reported: 1 Unresectablity: 4**

**Deceased before treatment: 1 Failure to registration: 4**

Final Randomisation N= 184

**Group A:** Standard treatment N= 95 (ITT)

**Group B:** Experimental group N=89 (ITT)

Surgery: 95 patients

Cytoreduction and HIPEC (89)

HIPEC ( Mitomycin C 15 mg/m2), 60 minutes (89)

Adjuvant systemic chemotherapy (local protocol) < 12 weeks after resection. ( 76 per protocol)

Adjuvant systemic chemotherapy (local protocol),

< 12 weeks after resection (75 pe rprotocol)

36 months follow-up

Efficacy and safety of Intraoperative Hyperthermic Intraperitoneal Chemotherapy (HIPEC) of Locally Advanced Colorectal Cancer

Long term analysis (36 months) of a prospective cohort study

**Contents**

Otero-Ferrer José Luis (PhD)

Biostatech – Advice, Training & Innovation in Biostatistics Ltd.

November 08, 2025

1. [Preamble 2](#_TOC_250015)
2. [Methods 2](#_TOC_250014)
3. [Results 2](#_TOC_250013)
   1. [Baseline demographic and clinical characteristics 2](#_TOC_250012)
   2. [Follow-up time 2](#_TOC_250011)
   3. [Overall survival 2](#_TOC_250010)
   4. [Disease-free survival 3](#_TOC_250009)
      1. [Sensibility analysis 5](#_TOC_250008)
      2. [Subgroup analysis 5](#_TOC_250007)
   5. [Loco-regional control 7](#_TOC_250006)
      1. [Sensibility analysis 7](#_TOC_250005)
      2. [Subgroup analysis 8](#_TOC_250004)
   6. [Recurrence type 10](#_TOC_250003)
4. [Supplementary material 11](#_TOC_250002)
   1. [Kaplan-Meier curve of the follow-up time 11](#_TOC_250001)

[References 11](#_TOC_250000)

# Preamble

Dataset (*hipect4 base madurada.sav*) contain 185 patients. Patient 270-3 was excluded from the analysis. The variable *lt_fallecido* was NA. According to the study first analysis, 184 patients were included in the analysis.

# Methods

The median follow-up time was estimating using the reverse Kaplan-Meier method (Xue et al. 2017). The endpoints was overall survival (OS), disease-free survival (DFS) and loco-regional control (LC) survival. The primary endpoint was LC. The secondary endpoints were DFS and OS. Kaplan-Meier survival analysis with log rank test will be used to compare the two study groups. Every hypothesis contrasts will be two-sided. A p-value of 0.05 will be considered statistically significant. Proportional hazard assumption will be tested using Schoenfeld residuals. Additionally, Restricted Mean Survival Time (RMST) and permutation test (Horiguchi and Uno 2020b) will be calculated at 12,24 and 36 months. The number of permutations was 10^4. The method selected was averaging RMSTs derived from Methods 2 and 3. Method 2 extending the survival curve to tau, and Method 3 switching the last censored observation to the event observation. RMST avoids the proportionality issues related to the Cox model (Royston and Parmar 2011).

Sensibility analysis were performed. The variables *perforacion*, *Well_differentiated*, *Poorly_differentiated*, *diferenciacion* and *localizacion* were included as a covariables in the analysis. Hazard ratios are presented with 95% confidence intervals. Well and poorly differentiated were created from the variable *diferenciacion*. Mucinous was created from the variable *tipohistol*. N0, N1, N2 were created from the variable *N*. pT4, pT4a, pT4b were created from the variable *T*. Adyuvancia was created from the variable *Adyuvancia* and when it is true represents the per-protocol population. Additionally, analysis were performed by subgroups (T4 and per protocol populations).

Fisher’s Exact test was used to compare recurrence types (*lt_tiporecid* ) between HIPEC groups.

Analysis were performed using R software (R Core Team 2024). Additional available CRAN packages were used to perform graphical (Wickham 2016; Sjoberg et al. 2024) and statistical analysis (Therneau 2024; Horiguchi and Uno 2020a; Uno et al. 2022; Heinzen et al. 2021).

# Results

## Baseline demographic and clinical characteristics

The study included 184 patients. In the following table are shown the baseline demographic and clinical characteristics of the patients (Table 1).

## Follow-up time

The median follow-up and survival times were estimating using the reverse Kaplan-Meier method. Table 2 shows the follow-up time of the patients included in the study.

The variable *lt_seguimiento* was the time to follow-up and the variable *lt_fallecido* was the event. 96% (95.94) of the patients were followed up to 36 months.

## Overall survival

The variable *lt_superv* was the time to death/follow-up, the variable *lt_fallecido* was the event and the variable *hipec* was the treatment group. Figure 1 shows the Kaplan-Meier curve of the overall survival.

The Hazard Ratio (95% CI) was 0.99 ( 0.42 - 2.33 ). The HR was non significant (pvalue=0.977). Goodness of fit was stablish using the Schoenfeld residuals test (pvalue=0.734).

RMST at 12, 24 and 36 months were calculated:

- The RMST (95% CI) at 12, 24 and 36 months of the Only Surgery group were 11.55 (11.15 - 11.95),

22.78 (21.83 - 23.72) and 33.64 (32.08 - 35.2) months, respectively.

Table 1: Baseline demographic and clinical characteristics of the patients included in the study. The table includes the number of missing values, mean and standard deviation for continuous variables, and the number and percentage for categorical variables.

| Surgery only (N=95) | | HIPEC (N=89) | Total (N=184) |
| --- | --- | --- | --- |
| sexo | |  |  |
| - Hombre | 55 (57.9%) | 56 (62.9%) | 111 (60.3%) |
| - Mujer | 40 (42.1%) | 33 (37.1%) | 73 (39.7%) |
| edad | | | |
| - Mean (SD) | 62.27 (10.66) | 60.62 (9.05) | 61.47 (9.92) |
| - Median (Range) | 65.00 (35.00, 85.00) | 61.00 (36.00, 76.00) | 63.00 (35.00, 85.00) |
| asa_score3 | | | |
| - N-Miss | 2 | 1 | 3 |
| - No | 65 (69.9%) | 60 (68.2%) | 125 (69.1%) |
| - Sí | 28 (30.1%) | 28 (31.8%) | 56 (30.9%) |
| Adyuvancia | | | |
| - No | 17 (17.9%) | 17 (19.1%) | 34 (18.5%) |
| - Sí | 78 (82.1%) | 72 (80.9%) | 150 (81.5%) |
| pT4 | | | |
| - No | 32 (33.7%) | 25 (28.1%) | 57 (31.0%) |
| - Sí | 63 (66.3%) | 64 (71.9%) | 127 (69.0%) |

Table 2: Follow-up time of the patients included in the study. The table includes the number of missing values, mean and standard deviation for continuous variables, and the median and range for categorical variables.

Surgery only (N=95) HIPEC (N=89) Total (N=184)

lt_seguimiento

| - N-Miss | 3 | 3 | 6 |
| --- | --- | --- | --- |
| - Mean (SD) | 33.90 (6.96) | 33.81 (6.45) | 33.86 (6.70) |

- Median (Range) 36.00 (1.00, 36.00) 36.00 (6.00, 36.00) 36.00 (1.00, 36.00)

- The RMST (95% CI) at 12, 24 and 36 months of the HIPEC group were 11.62 ( 11.26 - 11.97 ), 22.83

(21.9 - 23.76) and 33.45 (31.84 - 35.06) months, respectively.

- Differences between groups (HIPEC-Only surgery) in RMST at those time points were 0.071 (pvalue=0.787), 0.051 (pvalue=0.944), -0.193 (pvalue=0.868) months, respectively.

## Disease-free survival

The variable *DFS* was the time to recurrence/follow-up, the variable *RECID* was the event and the variable

*hipec* was the treatment group. Figure 2 shows the Kaplan-Meier curve of the disease-free survival.

The Hazard Ratio (95% CI) was 0.76 ( 0.42 - 1.4 ). The HR was non significant (pvalue=0.382). Goodness of fit was stablish using the Schoenfeld residuals test (pvalue=0.612).

RMST at 12, 24 and 36 months were calculated:

- The RMST (95% CI) at 12, 24 and 36 months of the Only Surgery group were 10.94 (10.37 - 11.51),

20.88 (19.48 - 22.28) and 29.96 (27.67 - 32.26) months, respectively.

- The RMST (95% CI) at 12, 24 and 36 months of the HIPEC group were 11.44 ( 11.04 - 11.85 ), 21.85

(20.71 - 23) and 31.42 (29.38 - 33.45) months, respectively.

100%


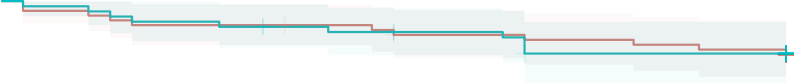

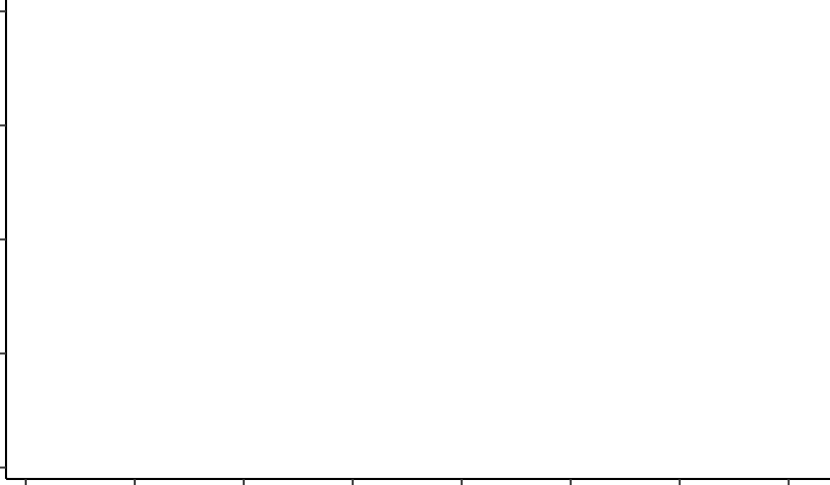


75%

50%

Overall survival

25%

0%

0 5 10 15 20 25 30 35

Time, in months

|  | At Risk (Events) |  | 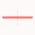 Surgery only | 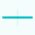 | HIPEC |  | |
| --- | --- | --- | --- | --- | --- | --- | --- |
| Surgery only | 95 (0) 92 (4) | 90 (5) | 89 (5) | 86 (7) | 85 (8) | 84 (9) | 83 (10) |
| HIPEC | 89 (0) 87 (3) | 85 (5) | 82 (6) | 79 (6) | 74 (10) | 74 (10) | 74 (10) |

Figure 1: Kaplan-Meier survival curves comparing overall survival between different HIPEC (Hyperthermic Intraperitoneal Chemotherapy) treatment groups. The x-axis represents time in months, and the y-axis represents the probability of overall survival. The plot includes confidence intervals and censor marks, with the risk table displaying the number of patients at risk and cumulative events at each time point.

100%


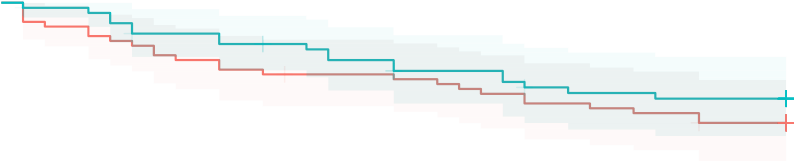

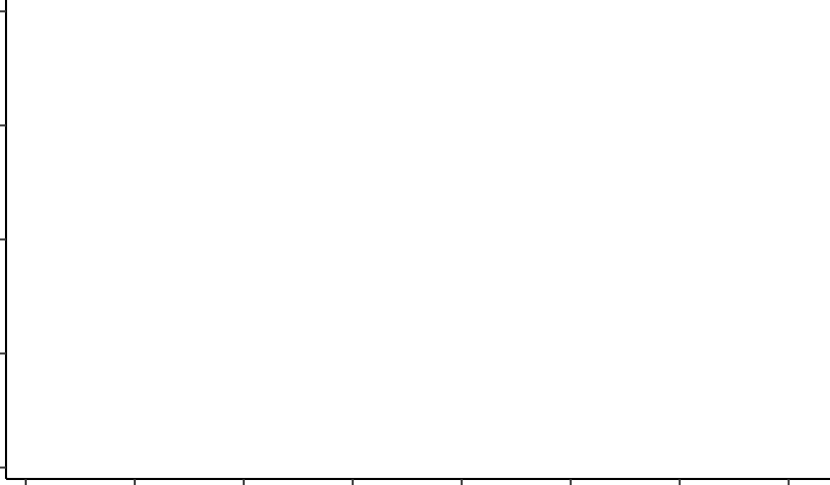


75%

Disease−free survival

50%

25%

0%

0 5 10 15 20 25 30 35

Time, in months

|  | At Risk (Events) | 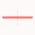 | Surgery only | 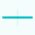 | HIPEC |  | |
| --- | --- | --- | --- | --- | --- | --- | --- |
| Surgery only | 95 (0) 88 (8) | 83 (14) | 79 (15) | 78 (17) | 73 (21) | 71 (23) | 68 (25) |
| HIPEC | 89 (0) 86 (4) | 81 (8) | 76 (11) | 70 (13) | 66 (16) | 65 (18) | 64 (18) |

Figure 2: Kaplan-Meier survival curves comparing disease-free survival between different HIPEC (Hyperther-mic Intraperitoneal Chemotherapy) treatment groups. The x-axis represents time in months, and the y-axis represents the probability of disease-free survival. The plot includes confidence intervals and censor marks, with the risk table displaying the number of patients at risk and cumulative events at each time point.

- Differences between groups (HIPEC-Only surgery) in RMST at those time points were 0.507 (pvalue=0.169), 0.972 (pvalue=0.299), 1.455 (pvalue=0.357) months, respectively.

### Sensibility analysis

Include the covariate in the model (Table 3), only main effect was included in each model.

Table 3: Univariate Cox proportional hazard regression analysis of loco-regional control. Covariates was includes in the model.

| covariate | HR | LCI | UCI | pvalue |
| --- | --- | --- | --- | --- |
| Perforation | 0.76 | 0.42 | 1.40 | 0.379 |
| Well differentiated | 0.79 | 0.43 | 1.47 | 0.459 |
| Poorly differentiated | 0.81 | 0.44 | 1.51 | 0.511 |
| Right colon | 0.76 | 0.42 | 1.40 | 0.386 |
| Left colon | 0.79 | 0.43 | 1.46 | 0.457 |
| Mucinous | 0.77 | 0.42 | 1.40 | 0.388 |
| pT4 | 0.71 | 0.39 | 1.31 | 0.274 |
| pT4a | 0.75 | 0.41 | 1.37 | 0.343 |
| pT4b | 0.76 | 0.41 | 1.39 | 0.372 |
| N0 | 0.73 | 0.40 | 1.33 | 0.302 |
| N1 | 0.75 | 0.41 | 1.38 | 0.356 |
| N2 | 0.76 | 0.42 | 1.40 | 0.383 |
| With adjuvant therapy | 0.77 | 0.42 | 1.40 | 0.387 |

In the following figure (figure 3) is shown the forest plot of the univariate hazard ratios estimation of the disease-free survival when each covariates is included in the model.

All the covariates were non-significant in the univariate analysis.

### Subgroup analysis

Subgroup analysis was performed. The hazard ratios of the subgroups T4 stage and adjuvant therapy were calculated (table 4 and figure 4).

Table 4: Univariate Cox proportional hazard regression subgroup analysis of disease free survival.

| subgroup | HR | LCI | UCI | pvalue |
| --- | --- | --- | --- | --- |
| With adjuvant therapy | 0.53 | 0.26 | 1.06 | 0.072 |
| T4 stage | 0.54 | 0.27 | 1.07 | 0.079 |

All subpopulations were non-significant in the univariate analysis.

RMST at 12, 24 and 36 months were calculated for the subgroup adyuvant therapy:

- - - - The RMST (95% CI) at 12, 24 and 36 months of the Only Surgery group were 10.85 (10.21 - 11.48),

20.55 (18.95 - 22.15) and 29.3 (26.68 - 31.92) months, respectively.

- - - - The RMST (95% CI) at 12, 24 and 36 months of the HIPEC group were 11.61 ( 11.22 - 12 ), 22.44

(21.35 - 23.54) and 32.5 (30.52 - 34.48) months, respectively.

- - - - Differences between groups (HIPEC-Only surgery) in RMST at those time points were 0.764 (pvalue=0.047), 1.894 (pvalue=0.064), 3.202 (pvalue=0.057) months, respectively.

RMST at 12, 24 and 36 months were calculated for the subgroup T4 stage:

With adjuvant therapy


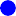

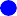

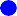

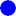

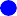

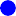

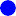

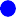

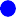

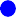

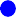

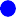

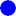


N2 N1 N0

pT4b pT4a pT4 Mucinous

Left colon Right colon

Poorly differentiated Well differentiated

Perforation

0.5 1.0 1.5

Hazard Ratio

Figure 3: Forest plot of the univariate Cox proportional hazard regression analysis of loco-regional control. The x-axis represents the hazard ratio, and the y-axis represents the covariates included in the model. The plot includes confidence intervals and the hazard ratio of the model. The dashed black line represents the null hypothesis (HR=1). The dashed blue line represents the hazard ratio of the model.

T4 stage

With adjuvant therapy

0.5 1.0

Hazard Ratio

Figure 4: Forest plot of the subgroup analysis of disease free survival (DFS). The x-axis represents the hazard ratio, and the y-axis represents the subgroups included in the model.

- - - - The RMST (95% CI) at 12, 24 and 36 months of the Only Surgery group were 10.4 (9.57 - 11.23), 19.55

(17.56 - 21.55) and 27.72 (24.52 - 30.91) months, respectively.

- - - - The RMST (95% CI) at 12, 24 and 36 months of the HIPEC group were 11.65 ( 11.29 - 12.01 ), 22.35

(21.21 - 23.49) and 31.87 (29.7 - 34.04) months, respectively.

- - - - Differences between groups (HIPEC-Only surgery) in RMST at those time points were 1.253 (pvalue=0.007), 2.797 (pvalue=0.02), 4.152 (pvalue=0.036) months, respectively.

## Loco-regional control

The variable *Controllocoregional* was the time to loco-regional control/follow-up, the variable *lt_recidperitoneal* was the event and the variable *hipec* was the treatment group. Figure 5 shows the Kaplan-Meier curve of the loco-regional control.

100%

75%

Loco−regional control

50%

25%

0%

0 5 10 15 20 25 30 35

Time, in months

|  | At Risk (Events) |  | Surgery only |  | HIPEC |  | |
| --- | --- | --- | --- | --- | --- | --- | --- |
| Surgery only | 95 (0) 91 (3) | 87 (5) | 85 (5) | 81 (7) | 77 (9) | 76 (10) | 75 (11) |
| HIPEC | 89 (0) 87 (0) | 85 (0) | 81 (1) | 77 (2) | 74 (2) | 74 (2) | 74 (2) |

Figure 5: Kaplan-Meier survival curves comparing loco-regional control between different HIPEC (Hyperther-mic Intraperitoneal Chemotherapy) treatment groups. The x-axis represents time in months, and the y-axis represents the probability of loco-regional control. The plot includes confidence intervals and censor marks, with the risk table displaying the number of patients at risk and cumulative events at each time point.

The Hazard Ratio (95% CI) was 0.19 ( 0.04 - 0.86 ). The HR was significant (pvalue=0.031). Goodness of fit was stablish using the Schoenfeld residuals test (pvalue=0.887).

RMST at 12, 24 and 36 months were calculated:

- The RMST (95% CI) at 12, 24 and 36 months of the Only Surgery group were 11.65 (11.33 - 11.98),

22.82 (21.95 - 23.7) and 33.5 (31.96 - 35.05) months, respectively.

- The RMST (95% CI) at 12, 24 and 36 months of the HIPEC group were 12 ( 12 - 12 ), 23.8 (23.53 -

24.08) and 35.51 (34.83 - 36.18) months, respectively.

- Differences between groups (HIPEC-Only surgery) in RMST at those time points were 0.346 (pvalue=0.038), 0.98 (pvalue=0.042) and 2.006 (pvalue=0.024) months, respectively.

### Sensibility analysis

Include the covariate in the model (Table 5 and figure 6, only main effect was included in each model.

Table 5: Univariate Cox proportional hazard regression analysis of loco-regional control. Covariates was includes in the model.

| covariate | HR | LCI | UCI | pvalue |
| --- | --- | --- | --- | --- |
| Perforation | 0.19 | 0.04 | 0.86 | 0.031 |
| Well differentiated | 0.20 | 0.04 | 0.90 | 0.036 |
| Poorly differentiated | 0.21 | 0.05 | 0.94 | 0.042 |
| Right colon | 0.19 | 0.04 | 0.86 | 0.031 |
| Left colon | 0.20 | 0.04 | 0.88 | 0.034 |
| Mucinous | 0.19 | 0.04 | 0.86 | 0.031 |
| pT4 | 0.17 | 0.04 | 0.77 | 0.022 |
| pT4a | 0.19 | 0.04 | 0.84 | 0.029 |
| pT4b | 0.19 | 0.04 | 0.86 | 0.031 |
| N0 | 0.17 | 0.04 | 0.77 | 0.022 |
| N1 | 0.19 | 0.04 | 0.85 | 0.030 |
| N2 | 0.19 | 0.04 | 0.84 | 0.029 |
| With adjuvant therapy | 0.18 | 0.04 | 0.83 | 0.028 |

### Subgroup analysis

Subgroup analysis was performed. The hazard ratios of the subgroups T4 stage and adjuvant therapy were calculated. Table 6 and figure 7 show the results.

Table 6: Subgroup analysis of loco-regional control. Covariates was includes in the model.

| subgroup | HR | LCI | UCI | pvalue |
| --- | --- | --- | --- | --- |
| With adjuvant therapy | 0.18 | 0.04 | 0.83 | 0.028 |
| T4 stage | 0.08 | 0.01 | 0.65 | 0.017 |

RMST at 12, 24 and 36 months were calculated for the subgroup adyuvant therapy:

- - - - The RMST (95% CI) at 12, 24 and 36 months of the Only Surgery group were 11.58 (11.2 - 11.97),

22.58 (21.53 - 23.62) and 32.97 (31.12 - 34.82) months, respectively.

- - - - The RMST (95% CI) at 12, 24 and 36 months of the HIPEC group were 12 ( 12 - 12 ), 23.77 (23.44 -

24.09) and 35.42 (34.62 - 36.22) months, respectively.

- - - - Differences between groups (HIPEC-Only surgery) in RMST at those time points were 0.418 (pvalue=0.028), 1.191 (pvalue=0.037) and 2.447 (pvalue=0.019) months, respectively.

RMST at 12, 24 and 36 months were calculated for the subgroup T4 stage:

- - - - The RMST (95% CI) at 12, 24 and 36 months of the Only Surgery group were 11.47 (10.99 - 11.96),

22.2 (20.89 - 23.5) and 32.16 (29.86 - 34.46) months, respectively.

- - - - The RMST (95% CI) at 12, 24 and 36 months of the HIPEC group were 12 ( 12 - 12 ), 23.9 (23.7 -

24.1) and 35.69 (35.09 - 36.29) months, respectively.

- - - - Differences between groups (HIPEC-Only surgery) in RMST at those time points were 0.529 (pvalue=0.01), 1.701 (pvalue=0.006), 3.529 (pvalue=0.001) months, respectively.

With adjuvant therapy


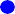

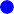

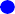

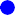

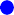

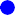

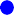

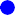

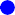

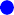

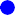

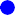

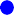


N2 N1 N0

pT4b pT4a pT4 Mucinous

Left colon Right colon

Poorly differentiated Well differentiated

Perforation

0.0 0.5 1.0 1.5

Hazard Ratio

Figure 6: Hazard ratio of the univariate Cox proportional hazard regression analysis of loco-regional control. The x-axis represents the hazard ratio, and the y-axis represents the covariates included in the analysis. The plot includes confidence intervals and a dotted black line indicating the null hypothesis (HR=1) and blue line the HR of the model.

T4 stage

With adjuvant therapy

0.0 0.5 1.0 1.5

Hazard Ratio

Figure 7: Hazard ratio of the subgroup analysis of loco-regional control. The x-axis represents the hazard ratio, and the y-axis represents the subgroups included in the analysis. The plot includes confidence intervals and a dotted black line indicating the null hypothesis (HR=1) and blue line the HR of the model.

## Recurrence type

The variable *Recidiva tipo* specify the recurrence type (categories: hematogenous (cerebral,hematogena,pul-monar), lymphatic (linfatica), peritoneal). Table 7 shows the recurrence type by treatment group (Fisher’s Exact Test).

Table 7: Recurrence type by treatment Group at 36-month follow-up.

| Surgery only (N=95) | | HIPEC (N=89) | Total (N=184) p value |
| --- | --- | --- | --- |
| lt_tiporecid | |  | 0.020 |
| - N-Miss | 72 | 70 | 142 |
| - Hematogenous | 11 (47.8%) | 14 (73.7%) | 25 (59.5%) |
| - Lymphatic | 1 (4.3%) | 3 (15.8%) | 4 (9.5%) |
| - Peritoneal | 11 (47.8%) | 2 (10.5%) | 13 (31.0%) |

Recurrence Type by Treatment Group

10

14

11

11

3

2

1

Number of Patients

RecurrenceType


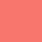
 Hematogenous
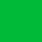
 Lymphatic

Peritoneal

5

0

Surgery only HIPEC

Treatment Group

Signficant differences were found between treatment groups (p-value<0.05).

# Supplementary material

## Kaplan-Meier curve of the follow-up time

100%

75%

50%

Percent followed

25%

0%

0 5 10 15 20 25 30 35

Time, in months

Figure 8: Kaplan-Meier plot illustrating the follow-up time of patients. The x-axis represents time in months, and the y-axis represents the percentage of patients followed. The plot includes confidence intervals and censor marks, with a dotted line indicating the median follow-up time.

# References

Heinzen, Ethan, Jason Sinnwell, Elizabeth Atkinson, Tina Gunderson, and Gregory Dougherty. 2021. *Arsenal: An Arsenal of ’r’ Functions for Large-Scale Statistical Summaries*. [https://CRAN.R-project.org/packag](https://CRAN.R-project.org/package%3Darsenal) [e=arsenal.](https://CRAN.R-project.org/package%3Darsenal)

Horiguchi, Miki, and Hajime Uno. 2020a. *survRM2perm: Permutation Test for Comparing Restricted Mean Survival Time*. [https://CRAN.R-project.org/package=survRM2perm.](https://CRAN.R-project.org/package%3DsurvRM2perm)

———. 2020b. “On Permutation Tests for Comparing Restricted Mean Survival Time with Small Sample from Randomized Trials.” *Statistics in Medicine* 39 (20): 2655–70. [https://doi.org/10.1002/sim.8565.](https://doi.org/10.1002/sim.8565)

R Core Team. 2024. *R: A Language and Environment for Statistical Computing*. Vienna, Austria: R Foundation for Statistical Computing. [https://www.R-project.org/.](https://www.R-project.org/)

Royston, Patrick, and Mahesh K B Parmar. 2011. “The Use of Restricted Mean Survival Time to Estimate the Treatment Effect in Randomized Clinical Trials When the Proportional Hazards Assumption Is in Doubt.” *Statistics in Medicine* 30 (19): 2409–21. [https://doi.org/10.1002/sim.4274.](https://doi.org/10.1002/sim.4274)

Sjoberg, Daniel D., Mark Baillie, Charlotta Fruechtenicht, Steven Haesendonckx, and Tim Treis. 2024.

*Ggsurvfit: Flexible Time-to-Event Figures*. [https://CRAN.R-project.org/package=ggsurvfit.](https://CRAN.R-project.org/package%3Dggsurvfit)

Therneau, Terry M. 2024. *A Package for Survival Analysis in r*. [https://CRAN.R-project.org/package=surv](https://CRAN.R-project.org/package%3Dsurvival) [ival.](https://CRAN.R-project.org/package%3Dsurvival)

Uno, Hajime, Lu Tian, Miki Horiguchi, Angel Cronin, Chakib Battioui, and James Bell. 2022. *survRM2: Comparing Restricted Mean Survival Time*. [https://CRAN.R-project.org/package=survRM2.](https://CRAN.R-project.org/package%3DsurvRM2)

Wickham, Hadley. 2016. *Ggplot2: Elegant Graphics for Data Analysis*. Springer-Verlag New York. [https://ggplot2.tidyverse.org.](https://ggplot2.tidyverse.org/)

Xue, Xiaonan, Ilir Agalliu, Mimi Y. Kim, Tao Wang, Juan Lin, Reza Ghavamian, and Howard D. Strickler. 2017. “New Methods for Estimating Follow-up Rates in Cohort Studies.” *BMC Medical Research*

*Methodology* 17 (1): 155. [https://doi.org/10.1186/s12874-017-0436-z.](https://doi.org/10.1186/s12874-017-0436-z)

**TITLE**

**“**Multicentre randomised clinical trial to evaluate the efficacy and safety of hyperthermic intraperitoneal chemotherapy (HIPEC) with Mitomycin C associated with surgery in the treatment of locally advanced colorectal carcinoma”

**EudraCT:** 2015-001801-15

**Protocol Code:** FCO-HIP-2015-01

**Version:** 1.2

**Date: 17.06.2015**

**DECLARATION OF CONFIDENTIALITY**

The information contained in this document is the property of the sponsor and is provided to the sponsor in confidence. It may not be disclosed to others without the written consent of the sponsor, except for the use that may be made to obtain the informed consent of the persons who will receive the investigational product, as well as in communications to the competent health authorities, Research Ethics Committees or those persons who will carry out the study.

# GENERAL INFORMATION

**A. Identification of the test:**

**Title:** “Multicentre randomised clinical trial to evaluate the efficacy and safety of hyperthermic intraperitoneal chemotherapy (HIPEC) with Mitomycin C associated with surgery in the treatment of locally advanced colorectal carcinoma”

**Nº EudraCT:** 2015-001801-15

**Protocol code:** FCO-HIP-2015-01

Version: 1.2

Date: 17/06/2015

**B. Identification of the promoter:**

FIBICO (Fundación para la Investigación Biomédica de Córdoba)

Hospital Universitario Reina Sofia

Avenida Menéndez Pidal s/n

14004 Córdoba

Teléfono: +34 957011040

**C. Monitoring Manager:**

FIBICO (Fundación para la Investigación Biomédica de Córdoba)

Unidad de Investigación Clínica y Ensayos Clínicos (UICEC)

Hospital Universitario Reina Sofía

Ed. Investigación Clínica. Nivel 1.

Avenida Menéndez Pidal s/n

14004 Córdoba

Teléfono: +34 957011040

**D. Pharmacovigilance Manager:**

FIBICO (Fundación para la Investigación Biomédica de Córdoba)

Unidad de Investigación Clínica y Ensayos Clínicos (UICEC)

Hospital Universitario Reina Sofía

Ed. Investigación Clínica. Nivel 1.

Avenida Menéndez Pidal s/n

14004 Córdoba

Teléfono: +34 957011040

**E. Coordinating Researcher:**

Dr. Álvaro Arjona Sánchez

Unidad de Gestión Clínica (UGC) General Surgery y Digestiva

Hospital Universitario Reina Sofía

Avenida Menéndez Pidal s/n

14004 Córdoba

Teléfono: 0043600328402

Fax: +34 957010949

**F. Clinical Research Ethics Committee:**

Research Ethics Committee of Cordoba

**G. Participating centres and research team:**

| **Researcher** | **Role** | **UGC / Service** | **Center** |
| --- | --- | --- | --- |
| **Alvaro Arjona Sánchez** | **PI/Coordinator** | **General Surgery/ Oncological Surgery Unit** | **H.U. Reina Sofía, Córdoba** |
| Sebastián Rufián Peña | Collaborator | General Surgery/ Oncological Surgery Unit | H.U. Reina Sofía, Córdoba |
| Francisco Cristóbal Muñoz Casares | Collaborator | General Surgery/ Oncological Surgery Unit | H.U. Reina Sofía, Córdoba |
| Angela Casado Adam | Collaborator | General Surgery/ Oncological Surgery Unit | H.U. Reina Sofía, Córdoba |
| Juan Manuel Sánchez Hidalgo | Collaborator | General Surgery/ Oncological Surgery Unit | H.U. Reina Sofía, Córdoba |
| César Díaz López | Collaborator | General Surgery/ Coloproctology Unit | H.U. Reina Sofía, Córdoba |
| Eva María Torres Tordera | Collaborator | General Surgery/ Coloproctology Unit | H.U. Reina Sofía, Córdoba |
| José Gómez Barbadillo | Collaborator | General Surgery/ Coloproctology Unit | H.U. Reina Sofía, Córdoba |
| Rubén García Martín | Collaborator | General Surgery / MIR | H.U. Reina Sofía, Córdoba |
| Enrique Aranda Aguilar | Collaborator | Medical Oncology | H.U. Reina Sofía, Córdoba |
| María Teresa Cano Osuna | Collaborator | Medical Oncology | H.U. Reina Sofía, Córdoba |
| Manuel Medina | Collaborator | Anatomical Pathology | H.U. Reina Sofía, Córdoba |
| Teresa Caro Cuenca | Collaborator | Anatomical Pathology | H.U. Reina Sofía, Córdoba |
| Cruces Garzas | Collaborator | Pharmacy | H.U. Reina Sofía, Córdoba |
| **Pedro Barrios Sánchez** | **PI** | **General Surgery** | **Hospital de Sant Joan Despí Moises Broggi, Barcelona** |
| Doménico Sabia | Collaborator | General Surgery | Hospital de Sant Joan Despí Moises Broggi, Barcelona |
| Isabel Ramos | Collaborator | General Surgery | Hospital de Sant Joan Despí Moises Broggi, Barcelona |
| Oriol Crusellas Mañas | Collaborator | General Surgery | Hospital de Sant Joan Despí Moises Broggi, Barcelona |
| **Gloria Ortega Pérez** | **PI** | **General Surgery** | **MD. Anderson, Madrid** |
| Santiago González Moreno | Collaborator | General Surgery | MD. Anderson, Madrid |
| Oscar Alonso Casado | Collaborator | General Surgery | MD. Anderson, Madrid |
| Sara Encinas García | Collaborator | Medical Oncology | MD. Anderson, Madrid |
| Alejandro Rojo Sebastián | Collaborator | Anatomical Pathology | MD. Anderson , Madrid |
| **Luis González Bayón** | **IP** | **General Surgery** | **H. U. Gregorio Marañón, Madrid** |
| Wenceslao Vásquez Jiménez | Collaborator | General Surgery | H. U. Gregorio Marañón, Madrid |
| Jose Manuel Asencio | Collaborator | General Surgery | H. U. Gregorio Marañón, Madrid |
| Aitana Calvo | Collaborator | Medical Oncology | H. U. Gregorio Marañón, Madrid |
| Ana Poza | Collaborator | Radiology | H. U. Gregorio Marañón, Madrid |
| Isabel Peligros Patóloga | Collaborator | Anatomical Pathology | H. U. Gregorio Marañón, Madrid |
| **Pedro Villarejo Campos** | **IP** | **General Surgery** | **H.U. Ciudad Real.** |
| Jesús Martín Fernández | Collaborator | General Surgery | H.U. Ciudad Real |
| David Padilla Valverde | Collaborator | General Surgery | H.U. Ciudad Real |
| Susana Sánchez García | Collaborator | General Surgery | H.U: Ciudad Real |
| Lucia González López | Collaborator | Anatomical Pathology | H.U: Ciudad Real |
| Juana María Cano Cano | Collaborator | Medical Oncology | H.U. Ciudad Real |
| Bartolomé López Viedma | Collaborator | Digestive | H.U. Ciudad Real |
| José Villanueva Liñán | Collaborator | Radiology | H.U. Ciudad Real |
| **Juan Torres Melero** | **IP** | **General Surgery/Oncological Surgery Unit** | **H. U. Torrecárdenas, Almería** |
| Francisco Javier Velasco Albendea | Collaborator | Anatomical Pathology | H. U. Torrecárdenas, Almería |
| Maria del Mar Berenguel Ibáñez | Collaborator | Anatomical Pathology | H. U. Torrecárdenas, Almería |
| Piedad Reche | Collaborator | Medical Oncology | H. U. Torrecárdenas, Almería |
| Begoña Medina Magan | Collaborator | Medical Oncology | H. U. Torrecárdenas, Almería |
| Angel Reina Duarte | Collaborator | General Surgery/ Coloproctology Unit | H. U. Torrecárdenas, Almería |
| Isabel Blesa Carrillo | Collaborator | General Surgery/Coloproctology Unit | H. U. Torrecárdenas, Almería |
| Miguel Angel Lorenzo Liñan | Collaborator | General Surgery/ Oncological Surgery Unit | H. U. Torrecárdenas, Almería |
| **Rafael Morales** | **IP** | **General Surgery /Oncological Surgery Unit** | **Hospital Son Espases, Palma de Mallorca** |
| Judit Perez Celada | Collaborator | General Surgery /Oncological Surgery Unit | Hospital Son Espases, Palma de Mallorca |
| Mónica Guillot | Collaborator | Oncology | Hospital Son Espases, Palma de Mallorca |
| Hermini Manzano | Collaborator | Oncology | Hospital Son Espases, Palma de Mallorca |
| Margarita Gamundi | Collaborator | General Surgery/ Coloproctology Unit | Hospital Son Espases, Palma de Mallorca |
| Miriam Fernández | Collaborator | General Surgery/ Coloproctology Unit | Hospital Son Espases, Palma de Mallorca |
| Joseba Olea | Collaborator | General Surgery/ Coloproctology Unit | Hospital Son Espases, Palma de Mallorca |
| Gonzalo Martín | Collaborator | General Surgery/ Coloproctology Unit | Hospital Son Espases, Palma de Mallorca |
| Elena Usamentiaga | Collaborator | Radiology | Hospital Son Espases, Palma de Mallorca |
| Josefa Lloret | Collaborator | Radiology | Hospital Son Espases, Palma de Mallorca |
| Carmen de Juan | Collaborator | Radiology | Hospital Son Espases, Palma de Mallorca |
| Isabel Amengual | Collaborator | Anatomical Pathology | Hospital Son Espases, Palma de Mallorca |
| Ana Forteza | Collaborator | Anatomical Pathology | Hospital Son Espases, Palma de Mallorca |
| **Joaquín Carrasco Campos** | **IP** | **General Surgery/ Oncological Surgery Unit** | **Hospital Carlos Haya, Málaga** |
| Javier Moreno Ruiz | Collaborator | General Surgery/ Oncological Surgery Unit | Hospital Carlos Haya, Málaga |
| Alberto Titos García | Collaborator | General Surgery/ Oncological Surgery Unit | Hospital Carlos Haya, Málaga |
| **Alberto Gutiérrez Calvo** | **IP** | **General Surgery/Peritoneal Carcinomatosis Unit** | **H. U. Príncipe Asturias, Alcalá Henares** |
| Inmaculada Lasa Unzué | Collaborator | General Surgery/Peritoneal Carcinomatosis Unit | H. U. Príncipe Asturias, Alcalá Henares |
| Remedios Gómez Sanz | Collaborator | General Surgery/Peritoneal Carcinomatosis Unit | H. U. Príncipe Asturias, Alcalá Henares |
| Adela López García | Collaborator | General Surgery/Peritoneal Carcinomatosis Unit | H. U. Príncipe Asturias, Alcalá Henares |
| Manuel Díez Alonso | Collaborator | General Surgery/ Colorectal Unit | H. U. Príncipe Asturias, Alcalá Henares |
| José María Muguerza Huguet | Collaborator | General Surgery/ Colorectal Unit | H. U. Príncipe Asturias, Alcalá Henares |
| Pilar Hernández Juara | Collaborator | General Surgery/ Colorectal Unit | H. U. Príncipe Asturias, Alcalá Henares |
| Raquel Molina Villaverde | Collaborator | Medical Oncology | H. U. Príncipe Asturias, Alcalá Henares |
| Carolina Castillo Torres | Collaborator | Anatomical Pathology | H. U. Príncipe Asturias, Alcalá Henares |
| José Ignacio Busteros Moraza | Collaborator | Anatomical Pathology | H. U. Príncipe Asturias, Alcalá Henares |
| **Jose Miguel Del Pino Monzón** | **IP** | **Colorectal Surgery and Peritoneal Oncology Surgery Units.** | **H. U. Nuestra Señora de la Candelaria. Tenerife** |
| Guillermo Hernández Hernández | Collaborator | Colorectal Surgery and Peritoneal Oncology Surgery Units | H. U. Nuestra Señora de la Candelaria. Tenerife |
| Vanessa Concepción Martin | Collaborator | Colorectal Surgery and Peritoneal Oncology Surgery Units | H. U. Nuestra Señora de la Candelaria. Tenerife |
| **Alfonso García Fadrique** | **IP** | **Digestive Oncological Surgery Service** | **Instituto Valenciano de Oncology. Valencia** |
| Rafael Estevan Estevan | Collaborator | Digestive Oncological Surgery Service | Instituto Valenciano de Oncology. Valencia |
| María Caballero Soto | Collaborator | Digestive Oncological Surgery Service | Instituto Valenciano de Oncology. Valencia |
| Amparo Martínez Blasco | Collaborator | Digestive Oncological Surgery Service | Instituto Valenciano de Oncology. Valencia |
| **Enrique Boldo Roda** | **IP** | **General Surgery /Oncological Surgery Unit** | **Hospital Provincial Castellón** |
| Isabel Busquier Fernandez | Collaborator | General Surgery /Oncological Surgery Unit | Hospital Provincial Castellón |
| Rafael Lozoya Albacarx | Collaborator | General Surgery /Oncological Surgery Unit | Hospital Provincial Castellón |
| Araceli Mayol Oltra | Collaborator | General Surgery /Oncological Surgery Unit | Hospital Provincial Castellón |
| **Bruno Camps** | **IP** | **General Surgery/ Oncological Surgery Unit** | **Hospital Clínico, Valencia** |
| **Estibalitz Pérez Viejo** | **IP** | **General Surgery/ Oncological Surgery Unit** | **H.U. Fuenlabrada** |
| Fernando Pereira | Collaborator | General Surgery/ Oncological Surgery Unit | H.U. Fuenlabrada |
| **Emilio Vicente** | **IP** | **General Surgery/ Oncological Surgery Unit** | **Clínica San Chinarro, Madrid** |
| Eduardo Díaz | Collaborator | General Surgery/ Oncological Surgery Unit | Clínica San Chinarro, Madrid |
| Isabel Fabra | Collaborator | General Surgery/ Oncological Surgery Unit | Clínica San Chinarro, Madrid |
| Yolanda Quijano | Collaborator | General Surgery/ Oncological Surgery Unit | Clínica San Chinarro, Madrid |

# SUMMARY OF PROTOCOL

1. **Development phase:**

Phase III

**1.1 Study Disease:** Colon and Rectal Cancer

**1.2 Study objectives:**

- - 1. **Main objective:**

To evaluate the efficacy of hyperthermic intraperitoneal chemotherapy (HIPEC) with Mitomycin C associated with extended cytoreductive surgery in the treatment of locally advanced colorectal carcinoma (pT4).

- - 1. **Secondary objectives:**

1) To evaluate the effect on overall survival (OS) of the addition of HIPEC with Mitomycin C to extended surgery at 12 months and 3 years.

2) To evaluate the effect on disease-free survival (DFS) of the addition of HIPEC with Mitomycin C to extended surgery at 12 months and 3 years.

3) To evaluate the safety (morbidity and mortality) of the addition of HIPEC to extended surgery for pT4 colorectal carcinoma.

**1.3. Study design:**

Multicentre, randomised, open-label, multicentre clinical trial to evaluate the efficacy and safety of hyperthermic intraperitoneal chemotherapy (HIPEC) with mitomycin C associated with extended cytoreductive surgery in the treatment of locally advanced colorectal carcinoma.

**1.4 Description of treatment groups:**

**Experimental Group:** Cytoreductive Surgery + Target Organ Surgery + HIPEC

**Control Group:** Cytoreductive surgery + target organ surgery + HIPEC

**1.5. Number of patients / Assignment to treatment:**

N= 200 Assignment to treatment 100/100.

**1.6. Variables:**

**1.6.1 Effectiveness:**

**- Main variable:**

Locoregional control (LC) in months and locoregional disease control rate (LC %) at 12 months, 3 years.

**- Secondary variables:**

1) Peri- and post-operative morbidity using the adverse event classification system “CTCAE” and peri-operative mortality, up to 30 and 90 days post-intervention.

2) Overall survival (OS) in months and survival rate (% OS at 12 m, 3 years).

3) Disease-free survival (DFS) in months and disease-free period rate (% DFS at 12 m, 3 years).

**1.6.2 Safety:**

The safety of both treatment groups will be assessed as a secondary endpoint as follows:

- Peri- and postoperative morbidity using CTCAE adverse event classification system and peri-operative mortality, up to 30 and 90 days post-intervention.
- Incidence and severity of adverse events (AE).

**1.7. Target population:**

Patients diagnosed with locally advanced colorectal cancer

**1.7.1 Selection criteria**

Patients will be prospectively included in the study if they meet ALL inclusion criteria and NO exclusion criteria.

**Inclusion criteria:**

1) Patients of both sexes, aged >18 years and <75 years.

2) Colon Adenocarcinoma, sigma and rectum-sigmoid junction with cT4a/b according to the American Joint Committee on Cancer (AJCC) TNM 7th edition.

3) Nodal extension: N0, the presence of N1/2 according to AJCC 7th edition TNM is allowed as long as they can be resectable.

4) Metastatic extension: M0.

5) Karnofsky Index >70 or Performance status ≤2.

6) Informed consent properly completed and signed.

**Exclusion criteria:**

1) Presence of metastases (M1).

2) Unresectability criteria.

3) Urgent intervention due to obstruction or perforation.

4) Extraperitoneal rectal cancer (avoiding alterations due to neoadjuvant treatment).

5) Coexistence of another malignant neoplastic disease.

6) Severely altered hepatic, renal or cardiovascular function.

7) Intolerance to treatment.

8) Administration of chemotherapy prior to the trial (neoadjuvant treatment is ruled out).

9) Women in gestational period or breastfeeding.

**1.7.2 Withdrawal criteria**

- 1. Withdrawal of the patient's informed consent.
  2. Deviation from the protocol. If deemed clinically appropriate by the investigators due to worsening of symptoms.
  3. Administrative decision taken by the investigators, sponsor or a regulatory authority.
  4. Loss of contact during follow-up.
  5. Serious adverse event (SAE) or clinically relevant adverse event.
  6. Unexpected serious adverse reaction (UASR).

**1.8. Tested treatment:**

Drug: Mitomycin C

Dose: 15 mg/M2/2 litres of infusion fluid

Schedule: Single dose

Pharmaceutical Form: Powder for solution for injection. The product, before reconstitution, is a blue-purple crystalline powder.

Route of administration: Intraperitoneal.

Pharmacotherapeutic group: Mitomycin

ATC code: L01DC03

**1.9. Treatment delivery:**

The Experimental Group will receive intraperitoneal Mitomycin C in hyperthermia in 4000cc of 1.5% dextrose for 60 minutes after completion of cytoreductive and target organ surgery.

**1.10. Study procedures:**

The total expected duration of each patient in the study will be 36 months and the following visits and procedures will take place:

Table 1. Schedule of visits.

| **Schedule of visits** | Selection | Basal | V1 | 1^st^ day PS | 2^nd^ day PS | 3^rd^ day PS | 7^th^ day PS | 1  month | 3 months | 6 months | 12 months | 18 months | 24 months | 36 months |
| --- | --- | --- | --- | --- | --- | --- | --- | --- | --- | --- | --- | --- | --- | --- |
| **Patient selection (After tumour commission)** | x |  |  |  |  |  |  |  |  |  |  |  |  |  |
| **Treatment and surgery** |  |  | x |  |  |  |  |  |  |  |  |  |  |  |
| **Pre-anaesthetic study** | x |  |  |  |  |  |  |  |  |  |  |  |  |  |
| **Physical examination** | x |  |  | x | x | x | x | x | x | x | x | x | x | x |
| **Informed consent** |  | x |  |  |  |  |  |  |  |  |  |  |  |  |
| **Tumour markers: CEA and CA 19.9** | x |  |  |  |  |  |  |  | x | x | x | x | x | x |
| **Biochemical analysis** | x |  |  | x | x | x | x | x |  |  |  |  |  |  |
| **Hemogram** | x |  |  | x | x | x | x | x |  |  |  |  |  |  |
| **Coagulation** | x |  |  | x | x | x | x | x |  |  |  |  |  |  |
| **Morbidity and mortality analysis** |  | x |  | x | x | x | x | x | x |  |  |  |  |  |
| **TC/RNM thoracic-abdominal-pelvic** | x |  |  |  |  |  |  |  | x |  | x |  | x | x |

**1.11. Statistical analysis:**

**1.11.1. Sample size determination**

The calculation of the clinical trial sample size is based on locoregional disease control rate (LC rate %) at 36 months after the intervention (primary endpoint). We have assumed that the proportion of patients in the treatment group with local disease control (LC rate %) at 36 months is 82% (peritoneal recurrence in 18% at 36 m). The proportion of patients in the control group with local disease control (LC rate %) at 36 months is 64% (peritoneal recurrence in 36% at 36 m).

With this estimate (error ɑ = 0.05, power = 0.80, two-tailed) the calculated sample size is 190 patients, 95 patients in each arm. Allowing for an approximate 5% loss of patients the final N is 200 patients.

**1.11.2. Statistical methods**

- Descriptive analysis for quantitative variables by calculating arithmetic means (m) and standard deviations (SD); and for qualitative variables by calculating percentage counts and proportions (%).
- Determination of goodness-of-fit to a normal distribution (normal data) using the Shapiro-Wilk test. Homogeneity of variances will also be tested using Levene's test.
- Comparison of mean values of quantitative variables between the two groups by Student's t-test (parametric test) or Mann-Whitney U-test (non-parametric test).
- -Comparison of proportions between the different groups by means of chi-square tests for contingency tables; in the case of 2 x 2 tables, the chi-square statistic with Yates correction will be used, and when any expected frequency is ≤ 5, Fisher's exact test will be applied.
- Association between quantitative variables by calculating Pearson's linear correlation coefficients (parametric test) or Spearman's correlation (non-parametric test), as appropriate.
- Survival curves will be studied by Kaplan-Meier method and comparison by Log-Rank to analyse the effect of the different factors on survival.

All hypothesis tests will be bilateral. In all statistical tests, values with a confidence level of 95% (p<0.05) will be considered ‘significant’.

**1.12. Work plan**

The total expected duration of the trial is 5 years from the inclusion of the first patient.

- Preparation of the protocol. Authorisation application to the AEMPS. Evaluation request to the CEIC of Reference: 2 months.
- Start of study: first patient expected to be included: June 2015.
- Patient recruitment/inclusion period: 24 months.
- Treatment period: each patient included will receive treatment for: 1 day.
- Follow-up period per patient: 36 months
- Completion of study: June 2020
- Statistical analysis and final report writing: September 2020.

# LIST OF TABLES

Table 1. Schedule of visits.

# LIST OF FIGURES

Figure 1: Study design

# Abbreviations

| **Abbreviations** | **Definition** |
| --- | --- |
| AE | Adverse events |
| SAE | Severe Adverse Events |
| AEMPS | Spanish Medicament Agency |
| GCP | Good Clinical Practise |
| CAFV | Andalusian Pharmacovigilance Center |
| CCEBRA | Coordinating Committee on the Ethics of Biomedical Research in Andalusia |
| CREC | Clinical Research Ethics Committee |
| DCN | Data Collection Notebook |
| CT | Clinical trial |
| ECG | Electrocardiogram |
| EMA | European Medicines Agency |
| HIPEC | Hyperthermic Intraperitoneal chemotherapy |
| ICH | International Conference of Armonization |
| IMIBIC | Biomedical Research Institute of Cordoba |
| SOP | Standard Operating Procedure |
| SAUR | Suspicious adverse and unexpected reactions |
| CT | Computed Tomography |

# ÍNDICE

[GENERAL INFORMATION 2](#_Toc188014028)

[SUMMARY OF PROTOCOL 9](#_Toc188014029)

[LIST OF TABLES 13](#_Toc188014030)

[LIST OF FIGURES 13](#_Toc188014031)

[GLOSARIO DE ABREVIATURAS 14](#_Toc188014032)

[ÍNDICE 15](#_Toc188014033)

[1. BACKGROUND AND JUSTIFICATION OF THE STUDY 19](#_Toc188014034)

[1.1 Background 19](#_Toc188014035)

[1.2 Pre-clinical and clinical experience: 20](#_Toc188014036)

[1.3 Justification of the study: 21](#_Toc188014037)

[2. OBJECTIVES OF THE STUDY 23](#_Toc188014038)

[2.2. Secondary objectives 23](#_Toc188014039)

[3. STUDY DESIGN 23](#_Toc188014040)

[3.1 Overview of the study design 23](#_Toc188014041)

[3.2 Study variables 23](#_Toc188014042)

[3.2.1 Effectiveness Variables 23](#_Toc188014043)

[3.2.2 Safety Variables 23](#_Toc188014044)

[3.3 Randomisation and blinding 24](#_Toc188014045)

[3.4 Participating centres 24](#_Toc188014046)

[3.5 Work plan 24](#_Toc188014047)

[3.6 Completion and discontinuation of the study 25](#_Toc188014048)

[4. STUDY POPULATION 27](#_Toc188014049)

[4.1 General characteristics 27](#_Toc188014050)

[4.2 Selection criteria 27](#_Toc188014051)

[4.2.1 Inclusion criteria: 27](#_Toc188014052)

[4.3 Criteria for termination and withdrawal of subjects 27](#_Toc188014053)

[4.4 Patient replacement 28](#_Toc188014054)

[4.5 Patient identification 28](#_Toc188014055)

[5. TREATMENT OF THE STUDY 28](#_Toc188014056)

[5.1 General description of the investigational medicinal product 28](#_Toc188014057)

[5.2 Manufacturing and labelling 29](#_Toc188014058)

[5.3 Shipping, storage and accounting 29](#_Toc188014059)

[5.4 Administration of research medication 30](#_Toc188014060)

[5.5 Dose Modification and Toxicity Management 30](#_Toc188014061)

[5.6 Risks and precautions in the use of research medication 30](#_Toc188014062)

[5.7 Concomitant medication 30](#_Toc188014063)

[5.7.1 Permitted concomitant medication 30](#_Toc188014064)

[5.7.2 Concomitant medication prohibited 31](#_Toc188014065)

[5.7.3 Interactions with other treatments 31](#_Toc188014066)

[5.8 Overdose 31](#_Toc188014067)

[5.9 Modification of treatment regimens due to adverse events (AA) 31](#_Toc188014068)

[5.11 Destruction of investigational medication 31](#_Toc188014069)

[6. STUDY PROCEDURES 31](#_Toc188014070)

[6.1 Procedures for the selection and inclusion of patients 31](#_Toc188014071)

[6.2. Procedures per visit 32](#_Toc188014072)

[6.3. Biological samples 36](#_Toc188014073)

[6.3.1. Blood samples. 36](#_Toc188014074)

[7. SAFETY ASSESSMENT / ADVERSE EVENTS 38](#_Toc188014075)

[7.1 Safety assessment 38](#_Toc188014076)

[7.2 Definition of adverse event 38](#_Toc188014077)

[7.3 Definition of Serious Adverse Events 39](#_Toc188014078)

[7.4 Definition of Adverse Reaction 40](#_Toc188014079)

[7.5 Definition of Serious and Unexpected Adverse Reactions 40](#_Toc188014080)

[7.6 Analytical anomalies and other abnormalities 41](#_Toc188014081)

[7.7 Assessment of intensity 41](#_Toc188014082)

[7.8 Assessment of causality 43](#_Toc188014083)

[7.9 Collection and Follow-up of Adverse Events 44](#_Toc188014084)

[7.10 Notification of Serious Adverse Events 44](#_Toc188014085)

[7.11 Expedited notification of SUARs to health authorities / CREC. 47](#_Toc188014086)

[7.12 Annual Safety Reports. 48](#_Toc188014087)

[7.13 Management of adverse events. 48](#_Toc188014088)

[7.14 Pregnancy. 48](#_Toc188014089)

[7.15 Deaths. 49](#_Toc188014090)

[8. STATISTICAL METHODOLOGY 49](#_Toc188014091)

[8.1 Data sets for analysis 49](#_Toc188014092)

[8.1.1 Intention to treat (ITT) population 49](#_Toc188014093)

[8.1.2 Per protocol population (PP) 49](#_Toc188014094)

[8.1.3 Safety data set 50](#_Toc188014095)

[8.2 Sample size determination 50](#_Toc188014096)

[8.3 Types of analysis 50](#_Toc188014097)

[9. ADMINISTRATIVE OBLIGATIONS 51](#_Toc188014098)

[9.1 Source documents 51](#_Toc188014099)

[9.2 Data collection and management 51](#_Toc188014100)

[9.3 Investigator's file/document retention 52](#_Toc188014101)

[9.4 Data Quality Assurance / Audits and Inspections 53](#_Toc188014102)

[9.5 Publication policy 53](#_Toc188014103)

[9.6 Study monitoring 54](#_Toc188014104)

[10. REGULATORY AND ETHICAL OBLIGATIONS 56](#_Toc188014105)

[10.1 Regulations 56](#_Toc188014106)

[10.2 Informed consent 56](#_Toc188014107)

[10.3 Confidentiality 57](#_Toc188014108)

[10.4 Good Clinical Practice (GCP) Responsibilities 59](#_Toc188014109)

[10.4.1 Sponsor's Responsibilities 59](#_Toc188014110)

[10.4.2 Responsibilities of the investigator 60](#_Toc188014111)

[10.5 Insurance 60](#_Toc188014112)

[10.6 Funding 60](#_Toc188014113)

[10.7 Conditions for amending the protocol 61](#_Toc188014114)

[10.8 Conditions for termination of the trial 61](#_Toc188014115)

[12. ANNEXES 64](#_Toc188014116)

[Annex 2. Example of study label. 67](#_Toc188014117)

[Annex 3. Serious Unexpected Adverse Reaction Reporting Occurring in Spain. 68](#_Toc188014118)

[Anexo 4. World Medical Association Declaration of Helsinki 70](#_Toc188014119)

# 1. BACKGROUND AND JUSTIFICATION OF THE STUDY

## *1.1 Background*

Colorectal cancer (CRC) is one of the most frequent neoplasms in our environment, with a growing trend over the last decade. According to data from the International Agency for Research on Cancer (IARC), in Spain in 2008, 16896 cases of colon and rectal cancer were diagnosed in men and 12005 in women, with an overall incidence of 45.7 cases per 100,000 inhabitants per year. This represents the second most frequent neoplasm in our country with a mortality rate of 21%, making it the second most frequent cause of death from cancer in our country (1)..

Prognosis of colon and rectal cancer is primarily established using the TNM or Dukes staging systems. These staging systems have their limitations, especially when the majority of patients are included in stages II and III, which are very heterogeneous categories. Other pathological variables have been investigated as prognostic tools in these stages, such as lymphatic and venous invasion, histological type, degree of differentiation, peritumoural lymphocytic infiltration, resection margin status, number of resected nodes, and tumour location. The most relevant histological feature (pT4) has also been recognised as an important prognostic factor in locoregional recurrence and survival (2). Shepherd et al. (3) established transserosal invasion with local peritoneal involvement (LPI) as a significant risk factor for peritoneal recurrence in colorectal cancer, establishing LPI type 3 (tumour present on the peritoneal surface with inflammatory reaction, mesothelial hyperplasia and/or erosion or ulceration) and LPI 4 (free tumour cells on the peritoneal surface with evidence of ulceration in the visceral peritoneum) as corresponding to pT4 in the TNM classification of equal risk of locoregional recurrence.

In this sense, if we evaluate the prognosis of patients with pT4 we observe that the overall survival at 5 years is 20% (91% for T1-2), obtaining a similar prognosis to those patients with N2 and M1. This leads us to consider the pT category by itself as a prognostic factor in survival. This is not the case for other typical prognostic factors such as lymphatic and venous invasion in specimen. Thus, the TNM categories are distorted in survival figures, because when we stratify on pT4, there is a variation in survival of stage II patients from 80% at 5 years, this decreases to 50% at 5 years when pT4 is present. In stage III the effect is similar, survival is 56% at 5 years, decreasing with the presence of pT4 to 30% at 5 years, and in N2 it is 37% at 5 years. In stage IV pT4 is not a prognostic factor in survival (2).

The addition of adjuvant therapy in stage II is controversial nowadays, being applicable to those patients with risk factors of which the one that conditions a worse prognosis is the presence of T4 category; thus several studies have shown that patients with T4N0M0 (II) have a worse prognosis than T1 N1 M0 (III), so it is not only lymph node invasion that is the main prognostic factor as postulated in the TNM and Dukes classifications. So far only a few retrospective studies consider the addition of adjuvant therapy to patients with stage II and pT4 based on 5-FU to be beneficial (2).

Hompes et al. (4) through their epidemiological study in T4 patients assessed the probability of peritoneal recurrence in 379 patients who underwent surgery for pT4 and stage II and III colorectal cancer, excluding T4 patients with distant metastases. For these patients, local recurrence and occurrence of peritoneal carcinomatosis was 15.6% at 12 months after surgery while for T3 it was 4.5% at 12 months (p = 0.008). Locoregional recurrence in patients with T3 was 20% at 5 years while for T4 it was 40% at 5 years of which 62% of them were the only site of metastases. It is this 26% of all T4 patients who could have benefited from adjuvant treatment with hyperthermic intraperitoneal chemotherapy (HIPEC) at the time of surgical resection, since survival in those patients who recurred with peritoneal carcinomatosis was only 6% at 5 years (4).

## *1.2 Pre-clinical and clinical experience:*

HIPEC gained its expansion thanks to the application and diffusion of peritonectomy procedures associated with HIPEC in the treatment of peritoneal metastases in colorectal cancer, which was developed by P.H. Sugarbaker (5). This procedure allows complete cytoreduction of all visible lesions in the abdomen, after which the application of intraperitoneal chemotherapy in hyperthermia allows high concentrations of chemotherapy to achieve destruction of microscopic lesions with minimal systemic adverse effects.

The application of this therapeutic modality has significantly improved survival in patients with colonic carcinomatosis from 23 months and 13% at 5 years with the best systemic chemotherapy to 62 months and 51% at 5 years, supporting these results to levels of evidence IA (6-8).

Nowadays, new concepts in the application of intraperitoneal chemotherapy are being studied, either as early postoperative administration (EPIC), as neoadjuvant or adjuvant chemotherapy or the application of intraperitoneal chemotherapy in hyperthermia as chemoprophylaxis or adjuvant chemotherapy in locally advanced tumours or tumours at high risk of developing carcinomatosis, a concept that is widespread in gastric carcinoma.

Along these lines, the French group at the Institute Gustave Roussy investigated (9) the use of second-look surgery and HIPEC for those patients at high risk of developing peritoneal carcinomatosis of colonic origin 12 months after the first operation. Of these, 56% had peritoneal disease at the time of second-look surgery and could be treated with cytoreductive surgery and HIPEC, increasing their survival compared to those who did not receive HIPEC. Following this preliminary study, the Prophylochip study was launched in France to compare standard follow-up with second-look surgery + HIPEC.

This same concept of adjuvant HIPEC application has also been studied more extensively for locally advanced gastric tumours with serosal involvement improving the prognosis of these patients (10).

Tentes and collaborators (11) conducted a study on the application of HIPEC with mitomycin C or oxaliplatin on 41 patients and EPIC on 63, with a 3-year survival of 100% in the HIPEC group, including T3 and T4 patients and finding a clear superiority of HIPEC in terms of survival over EPIC.

Sammartino and collaborators (12) propose in their study something similar, the application of HIPEC with oxaliplatin for T3 mucinous/T4 colorectal carcinoma and compare it with a control group with standard surgery. They enrolled only 25 patients in the experimental group with a recurrence at 36 months of 4% versus 22% in the control group, with the reservation that in the experimental group targeting surgery was performed (surgery extended to omentectomy, bilateral oophorectomy in postmenopausal women and appendectomy) and in the control group it was not performed.

The suggestion by Hompes and Collaboratores (4) and also by Baratti and Collaboratores (13) to add HIPEC as an adjuvant or in second look to risk groups is not new, but it has not been extensively studied, only a few pilot studies have been able to conclude survival benefits and especially safety of the treatment provided. In this regard, only a large prospective study by Noura and collaborators (14) explored the effects of HIPEC in patients with colorectal carcinoma with positive peritoneal lavage as prevention of locoregional recurrence, concluding that HIPEC with Mitomycin C appeared effective in preventing peritoneal recurrence and prolonging survival, achieving a reduction from 50% in the non-HIPEC group to 12% in the HIPEC group with no increase in morbidity and mortality.

## *1.3 Justification of the study:*

The aim of our study was to find an effective and safe treatment to prevent the appearance of peritoneal disease after tumour resection, increasing the locoregional control of the disease, disease-free period and survival in patients with cT4NxM0 colorectal carcinoma with the use of hyperthermic intraperitoneal chemotherapy (HIPEC) with mitomycin C and surgery extended to target organs, establishing the differences with the control group (surgery extended to target organs). Thus, HIPEC fulfils the expectation of a locally aggressive treatment with minimal side effects that has also provided clear benefits in terms of survival in patients with advanced cancer disease.

This would suggest the potential benefit of HIPEC as an adjuvant or chemoprophylaxis to improve disease-free survival and therefore overall survival in patients with locally advanced colorectal carcinoma (cT4) with little associated morbidity. This option is attractive for professionals who face peritoneal recurrences on a daily basis after extremely complex surgeries, but its application is difficult today due to the increase in economic and resource costs, which leads to the need for its administration and evaluation in clinical trials, These trials, in order to obtain adequate power and significance, need to be multicentre in nature, carried out in centres with specialised Oncological Surgery Units with extensive experience in peritoneal carcinomatosis that perform cytoreduction and HIPEC procedures. (15-18).

It is this group of Oncological Surgery Units focused on the treatment of disseminated peritoneal disease (GECOP Group under the auspices of the SEOQ) that is the field of implementation of the study we present. The GECOP group was created to respond to the need to find a union between those groups that embarked on the treatment of disseminated peritoneal disease with peritonectomy and HIPEC procedures, with each of the constituent Units becoming reference points for the referral of highly complex patients such as those requiring such treatment (digestive, gynaecological or peritoneal carcinomatosis). The application of HIPEC as an adjuvant in locally advanced carcinomas with risk of subsequent development of carcinomatosis is a topic that is always on the rise, both within our national GECOP group and in the international community where it is currently the trend in the various clinical trials launched by different advanced oncological surgery groups.

## *2.* *OBJECTIVES OF THE STUDY*

**2.1. Main objective**

To evaluate the efficacy of hyperthermic intraperitoneal chemotherapy (HIPEC) with Mitomycin C associated with extended cytoreductive surgery in the treatment of locally advanced colorectal carcinoma (cT4).

## *2.2. Secondary objectives*

1) To assess the effect on overall survival (OS) of the addition of HIPEC with Mitomycin C to extended surgery at 12 months and 3 years.

2) To evaluate the effect on disease-free survival (DFS) of the addition of HIPEC with Mitomycin C to extended surgery at 12 months and 3 years.

3) To assess the safety (morbidity and mortality) of adding HIPEC to extended surgery for cT4 colorectal carcinoma.

# 3. STUDY DESIGN

## *3.1 Overview of the study design*

Multicentre, randomised, open-label, multicentre clinical trial to evaluate the efficacy and safety of hyperthermic intraperitoneal chemotherapy (HIPEC) with Mitomycin C associated with extended cytoreductive surgery in the treatment of locally advanced colorectal carcinoma..

## *3.2 Study variables*

### 3.2.1 Effectiveness Variables

**Primary variable:**

Locoregional control (LC) in months and locoregional disease control rate (LC %) at 12 months, 3 years.

**Secondary variables:**

1) Peri- and postoperative morbidity using the CTCAE adverse events classification system and peri-operative mortality, up to 30 and 90 days post-intervention.

2) Overall survival (OS) in months and survival rate (% OS at 12 m, 3 years).

3) Disease-free survival (DFS) in months and rate of disease-free period (% DFS at 12 m, 3 years).

### 3.2.2 Safety Variables

The safety of both treatment groups will be assessed as a secondary objective in the following way:

- Peri- and postoperative morbidity using the CTCAE adverse event classification system and peri-operative mortality, up to 30 and 90 days post-intervention.
- Incidence and severity of adverse events (AEs).

## *3.3 Randomisation and blinding*

A total of 200 patients are planned to be included and divided into two groups:

- Treatment Group: 100 patients
- Control Group: 100 patients

Patients will be randomly assigned to one of the two treatment groups in a 1:1 ratio, using a randomisation list generated by a dedicated computer system.

This is an open study, so masking is not necessary. As this is an intraoperative treatment application, it is not possible to mask the patient or the doctor, due to the inherent characteristic of the surgical technique and to the fact that the patient systematically receives a hospital discharge report with a description of the surgical procedure carried out. Knowledge of the technique applied has no influence on the variables under study.

## *3.4 Participating centres*

The clinical trial will be conducted in 15 national centres.

## *3.5 Work plan*

The total planned duration of the trial is 60 months from the inclusion of the first patient:

- Preparation of the protocol. Authorisation application to the AEMPS. Evaluation request to the reference CEIC: 2 months.
- Start of the study: After obtaining authorisation from the AEMPS and a favourable opinion from the reference CEIC, the first patient is expected to be included in 2015.
- Patient recruitment/inclusion period: 2 years.
- Treatment period: each patient included will receive treatment in a single session.
- Follow-up period per patient: 36 months after completion of treatment.
- End of study (last contact of the last patient included): 2020.
- Statistical analysis and writing of the final report: two months after the last patient.

The total expected duration of each patient in the study is 36 months:

Each patient will make a total of 14 visits, in the following sequence (See Table 1):

● Selection period.

● Inclusion and randomisation period: baseline.

● Treatment period.

● Follow-up period: 1st, 2nd, 3rd, 4th and 7th postoperative days, 1, 3, 6, 12, 12, 18, 24 and 36 months.

The trial visits are described below:

- Screening visit (SV) (Day -15): Informing the patient about the study and obtaining informed consent. Assessment of inclusion and exclusion criteria. Clinical history and physical examination, vital signs, laboratory samples, pre-anaesthetic study.

- Baseline inclusion and randomisation visit (V0): Review of inclusion and exclusion criteria, randomisation

- Visit 1 (V1): surgical procedure and treatment.

- Follow-up period: A total of 12 visits (up to 36 months)

Visits V2-V6 (1st, 2nd, 3rd, 4th and 7th postoperative days): the first visits will be on an inpatient basis, where safety variables, laboratory analyses, etc. will be evaluated.

Visits V7-V13 (per month, 3m, 6m, 12m, 18m, 24m, 36m): clinical examination, laboratory samples, tumour markers and assessment by CT/MRI thoraco-abdominal imaging tests.

## *3.6 Completion and discontinuation of the study*

The trial should be terminated when the last patient enrolled in the trial has completed the last follow-up visit or withdraws from the trial for the reasons indicated in Section 4.3 ‘Criteria for Termination and Subject Withdrawal’.

If the trial is prematurely terminated or suspended, the sponsor should promptly inform the investigators, the participating investigator(s), the trial site(s), and the regulatory authority(ies) of the termination or suspension and the reasons for the termination or suspension. In addition, the sponsor should promptly inform the IRB/IEC and provide the IRB/IEC with the justification for termination or suspension as specified in the relevant legal requirements.

Whether the study is completed or prematurely terminated, the sponsor should ensure that trial reports are prepared and provided to the regulatory agencies as specified in the relevant regulatory requirements.

**Figure 1. Study design**

**
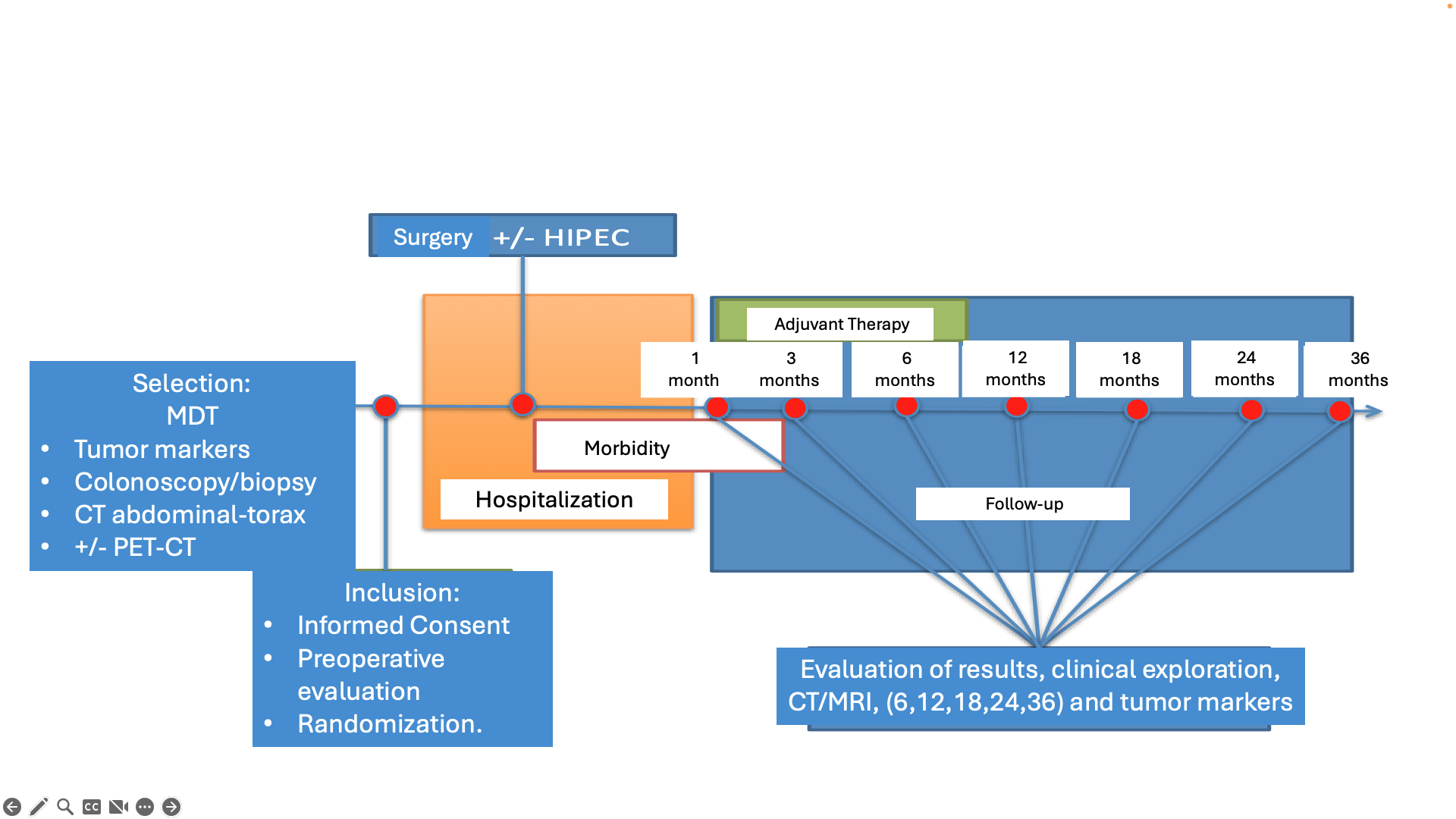
**

# 4. STUDY POPULATION

## *4.1 General characteristics*

Patients diagnosed with locally advanced adenocarcinoma of the colon and upper rectum.

## *4.2 Selection criteria*

Patients will be prospectively included in the study if they meet ALL inclusion criteria and NO exclusion criteria.

### 4.2.1 Inclusion criteria:

1) Patients of both sexes, aged >18 years and <75 years;

2) Adenocarcinoma of the colon, sigma and rectum-sigmoid junction with cT4a/b according to the American Joint Committee on Cancer (AJCC) TNM 7th edition.

3) Nodal extension: N0, the presence of N1/2 according to AJCC 7th edition TNM is allowed as long as they can be resectable.

4) Metastatic extension: M0.

5) Karnofsky Index >70 or Performance status ≤2.

6) Informed consent duty completed.

**4.2.2 Exclusion criteria:**

1) Presence of metastases (M1).

2) Presence of unresectability criteria.

3) Urgent intervention due to obstruction or perforation.

4) Extraperitoneal rectal cancer (avoiding alterations due to neoadjuvant treatment).

5) Coexistence of another malignant neoplastic disease.

6) Severely altered hepatic, renal or cardiovascular function.

7) Intolerance to treatment.

8) Administration of chemotherapy prior to the trial (neoadjuvant treatment is ruled out).

9) Women in the gestational period or breastfeeding.

## *4.3 Criteria for termination and withdrawal of subjects*

A subject is considered to have completed the trial when the subject makes the last scheduled follow-up visit.

Any subject who does not follow the trial procedure, has not been followed up, or for whom no further information is available since the date of withdrawal or last contact will be considered to have withdrawn from the trial.

The reasons for withdrawal will be analysed in full compliance with the principles of Bioethics, in terms of guaranteeing patients' rights and autonomous and informed decision.

Patients may withdraw at any time throughout the study, for any reason, without prejudice to future medical treatment.

Although patients may withdraw without having to explain the reason, as soon as a patient has decided to do so, investigators will attempt to contact subjects who do not return for scheduled visits or check-ups and establish that the patient's decision is an informed choice, and to ascertain to what extent the patient might be willing to continue to participate in the study in a limited way, e.g. whether he/she would be willing to continue to be contacted or seen, in order to obtain follow-up information.

In case of premature withdrawal, the investigator will conduct all examinations scheduled for the end-of-study visit.

Information relevant to the withdrawal will be documented in the CRF. The investigator shall indicate whether the decision to withdraw from the trial was made by the patient or by the investigator and shall indicate which of the following possible reasons for withdrawal is the reason for withdrawal:

1. Patient request and withdrawal of informed consent.
2. Infringement or deviation from the protocol.
3. If the investigators consider it appropriate, from the clinical point of view, due to a worsening of the patient's disease state.
4. Administrative decision taken by the investigators, sponsor or a regulatory authority.
5. Loss of contact during follow-up.
6. Unexpected serious adverse drug reaction (UASR).
7. Serious adverse event (SAE).
8. Any adverse or intercurrent event that is considered intolerable by the patient or incompatible with the continuation of the trial in agreement with the investigator.
9. Suspected pregnancy or positive pregnancy test result. Any suspected pregnancy should be followed immediately by a confirmatory serum pregnancy test.

A clear distinction should be made between subjects who withdraw from the study due to SAE/ UASR and those who withdraw for other reasons. The investigators will follow subjects who withdraw due to SAE/ UASR until the event has been resolved.

In case of withdrawal from the study, patients will be treated according to existing protocols and at the discretion of the investigator.

## *4.4 Patient replacement*

Recalled patients will not be replaced.

## *4.5 Patient identification*

All patients who have signed the informed consent form will receive a coding number, which will be used to identify them throughout the study.

Patients will be identified by a code that includes the site number followed by a chronological inclusion number for that site (XX-YY).

# 5. TREATMENT OF THE STUDY

## *5.1 General description of the investigational medicinal product*

Investigational medicinal product (IMP) is defined as ‘a pharmaceutical form of an active substance or placebo being studied or used as a reference in a clinical trial, including products with a marketing authorisation when used or combined (formulated or packaged) in a form different from the authorised form, or when used for an unauthorised indication, or for further information on the authorised form’.

In the present study, the IMP consists of Mitomycin. It is an antitumour antibiotic that is activated in tissues, behaving as an alkylating agent that disorganises deoxyribonucleic acid (DNA) in cancer cells by complexing with DNA and also acts by inhibiting cell division in cancer cells by interfering with DNA biosynthesis.

Drug: Mitomycin

Dose: 15 mg/ M2 /2 Litre of perfusion fluid (dextrose 1.5%)

Schedule: Single intraoperative dose.

Pharmaceutical Form: Powder for solution for injection. The product, before reconstitution, is a blue-purple crystalline powder.

Route of administration: Intraperitoneal.

Pharmacotherapeutic group: Mitomycin

ATC code: L01DC03

## *5.2 Manufacturing and labelling*

Mitomycin C will be manufactured and supplied by the Hospital Pharmacy Service of each participating site, following Good Manufacturing Practice (GMP).

The containers used for the trial will be identified by a label, according to current GMP, GCP guidelines and current national legal requirements (Annex 2)..

## *5.3 Shipping, storage and accounting*

The investigational medicinal product will be received by the investigator or pharmacist (if applicable) and they will be responsible for safe and proper handling and storage of the investigational product.

The IMP should be stored in the Pharmacy Department of each participating site, in a locked facility, with access limited to authorised site personnel, and under physical conditions that meet the specific requirements of the investigational product.

Updated temperature records should be maintained by the investigator or pharmacist to document proper storage during the course of the trial.

When requested by the investigator, the investigational product(s) should be prepared by the responsible personnel in the Pharmacy Service according to the current protocol, ensuring optimal safety conditions at all times. Once the medication has been prepared, it will be sent to the operating theatre for intraoperative administration.

The investigator should ensure that the IMP is administered only to patients participating in this study.

The IMP should not be used outside the context of this study protocol. The investigator or authorised personnel are required to document the receipt, dispensing and return of all IMPs received during this trial.

Medication should not be used beyond the expiry date printed on the outer container.

At the end of each patient's participation in the study, all remaining IMPs must be returned to the Sponsor for an accurate accounting of IMPs given and returned.

A record should be kept of receipt, use, return, loss or other disposition of the IMPs. Receipt forms should be signed by the investigator or, where appropriate, the pharmacist. The investigator or pharmacist, or other appropriately qualified person at the investigational site, should keep records of delivery to the site, inventory at the site, use by each patient, and return to the Sponsor of the IMPs. These records should include dates, quantities, lot numbers, and unique code numbers assigned to the IMP and patients. Investigators should keep records documenting that patients received the doses specified in the protocol. In addition, they must reconcile all IMPs received from the Sponsor. It is the investigator's responsibility to explain any discrepancies in the accounting of IFs.

All remaining IFs will be collected and returned to the Sponsor for destruction at the end of the study.

## *5.4 Administration of research medication*

Mitomycin C will be administered intraperitoneally intraoperatively in continuous perfusion by hyperthermia machine for 60 minutes at a temperature of 42-43ºC, in a 4000cc solution of 1.5% dextrose at a dose of 15 mg/M2/2L (HIPEC).

Once the surgical cytoreduction has been completed and before any digestive anastomosis is performed, intraperitoneal intraoperative hyperthermic chemotherapy (HIPEC) with Mitomycin C will be administered. The solution containing the cytostatic will be administered into the abdominal cavity (Mitomycin C at a dose of 15 mg/m2 per 2 litres of 1.5% dextrose solution) for 60 minutes. During the time of the perfusion, which will be maintained at an intra-abdominal temperature between 41-42°C by means of a heat exchanger, all internal anatomical structures of the peritoneal cavity will be uniformly exposed to the chemotherapy. Two perfusion pumps will deliver the chemotherapy solution with a high flow rate (1l/min) into the abdomen through two infusion catheters and extract it through four suction drains placed in the abdominal cavity (subdiaphragmatic and pelvic). Through a smoke evacuator, the air will be extracted under a plastic cover that will isolate the cavity in the form of a hood, thus avoiding possible contamination of the operating room air by cytotoxic aerosols. When the intraoperative perfusion is completed, all fluid is aspirated from the abdomen and the reconstructive phase of the surgery begins.

## *5.5 Dose Modification and Toxicity Management*

The dose will depend on the calculation of body surface area at a rate of 15mg/M2/2 litres. Patients with nephropathy, liver disease or women in the gestational period are not included, so no dose modification is necessary.

## *5.6 Risks and precautions in the use of research medication*

Potential adverse reactions and contraindications, warnings and precautions for use are listed in the Mitomycin C label.

## *5.7 Concomitant medication*

It is defined as any drug other than the investigational trial medication.

Concomitant treatments will be recorded in the patient's DCN and the name of the drug, total daily dose, route of administration, start and end dates, and reason for administration will be specified.

Patients receiving any prohibited concomitant medication or any medication in an unpermitted dose that cannot be discontinued or reduced will not be allowed to participate in the study.

### 5.7.1 Permitted concomitant medication

The following concomitant treatments are permitted during the study:

- Any chronic treatment of the patient shall be allowed.
- Thus, the administration of adjuvant therapy by means of schemes based on fluoropyrimidines (5-FU, Capecitabine), platinum (Oxaliplatin) and/or irinotecan will be allowed.
- Monoclonal antibodies such as bevacizumab and cetuximab.

### 5.7.2 Concomitant medication prohibited

The treatments prohibited during the entire study are:

- Neoadjuvant chemotherapy treatment.

### 5.7.3 Interactions with other treatments

The following treatments are not recommended during the entire active treatment phase:

- There are no

## *5.8 Overdose*

There is no known antidote for Mitomycin C overdose.

In case of overdose, the patient should be closely monitored and the toxicities manifested should be treated.

## *5.9 Modification of treatment regimens due to adverse events (AE)*

In case of AE, the impact on the patient and the relationship with the investigational medicinal product administered shall be assessed.

## *5.11 Destruction of investigational medication*

At the end of the trial, unused investigational products should be disposed of.

If destruction is performed at the trial site, the investigator should ensure that the materials are destroyed in compliance with applicable environmental regulations and site policy. Destruction should be properly documented.

# 6. STUDY PROCEDURES

## *6.1 Procedures for the selection and inclusion of patients*

Prior to any study activity, patients will be asked to read and sign an informed consent form that has been approved by a Clinical Research Ethics Committee and meets regulatory requirements. Patients will be given time to review any study-related information provided by the investigator. As part of the informed consent procedure, patients will be allowed to ask the investigator any questions about the potential risks and benefits of participating in the study.

To participate in the study, patients must meet ALL inclusion criteria, and NO exclusion criteria.

Once the selection criteria are confirmed and the patient is deemed eligible to participate in the study, he/she will be randomly assigned to a treatment group.

The investigator shall document in the CRF the fulfilment of the selection criteria for subjects considered for participation in the study. In addition, a list of inclusion and patient identification codes must be maintained.

## *6.2. Procedures per visit*

The following visits will take place during the trial:

**SELECTION VISIT (SC) (Day -15)**

The following assessments and studies will be performed:

- Inform patient about the trial and obtain written, signed and dated informed consent.

- Document demographic data

- Assessment of selection criteria

- Document patient's medical history

- Perform physical examination (including height, weight, vital signs)

- Blood sample to perform haemogram, biochemistry, coagulation, tumour markers (as per standard clinical practice)

- Radiological monitoring by thoracoabdominal-pelvic MRI/CT and/or PET (as per standard clinical practice)

- Check patient's current methods of birth control and perform serum pregnancy test on women of childbearing age (if applicable).

- Concomitant medications or non-pharmacological therapies, including reason for administration.

- Appointment for next visit.

**BASAL OR INITIAL VISIT (V0)**

The baseline visit can take place up to 15 days after the screening visit when all the results of the screening assessment are available.

The following assessments will be carried out:

- Hospital admission

- Reassessment of inclusion and exclusion criteria.

- Perform physical examination (including height, weight, vital signs)

- Trial enrolment, randomization and treatment allocation

- Document patient history

- Perform physical examination (including height, weight, vital signs)

- Blood sample

**VISIT 1 (Surgical intervention and treatment)**

- Surgical treatment and HIPEC

- Recording any AE

**VISIT 2 (1st postoperative day)**

- Clinical examination

- Blood test (biochemistry, haemogram and coagulation)

- Recording any adverse events

**VISIT 3 (2nd postoperative day)**

- Clinical examination

- Blood test (biochemistry, haemogram and coagulation)

- Recording any adverse events

**VISIT 4 (3rd postoperative day)**

- Clinical examination

- Blood test (biochemistry, haemogram and coagulation)

- Recording any adverse events

**VISIT 5 (4th postoperative day)**

- Clinical examination

- Blood test (biochemistry, haemogram and coagulation)

- Recording any adverse events

**VISIT 6 (7th postoperative day)**

- Clinical examination

- Blood test (biochemistry, haemogram and coagulation)

- Recording any adverse events

**VISIT AT HOSPITAL DISCHARGE**

Carried out by the principal investigator and collaborators on the hospital ward:

- Clinical examination

- Blood tests (biochemistry, haemogram and coagulation)

- Recording any adverse events

- Record of hospital stay (days)

- Record of postoperative morbidity

**VISIT 7 (1 month post-treatment)**

- Clinical examination

- Blood test (biochemistry, haemogram and coagulation)

- Recording any adverse events

**VISIT 8 (3 months post-treatment)**

- Clinical examination

- Tumour markers CEA and CA 19.9

- Recording any adverse events

**VISIT 9 (6 months post-treatment)**

- Clinical examination

- Tumour markers CEA and CA 19.9

- Abdominal thoracic CT/MRI

- Recording any adverse events

**VISIT 10 (12 months post-treatment)**

- Clinical examination

- Tumour markers CEA and CA 19.9

- Abdominal thoracic CT/MRI

- Recording any adverse events

**VISIT 11 (18 months post-treatment)**

- Clinical examination

- Tumour markers CEA and CA 19.9

- Abdominal thoracic CT/MRI

- Recording any adverse events

**VISIT 12 (24 months post-treatment)**

- Clinical examination

- Tumour markers CEA and CA 19.9

- Abdominal thoracic CT/MRI

- Recording any adverse events

**VISIT 13 (36 months post-treatment)**

- Clinical examination

- Tumour markers CEA and CA 19.9

- Abdominal thoracic CT/MRI

- Recording any adverse events

## *6.3. Biological samples*

### 6.3.1. Blood samples.

During the trial, several blood samples will be collected for haemogram, biochemistry, coagulation and serum levels of the tumour markers CEA and Ca19.9 (samples collected according to standard clinical practice).

These determinations will be performed at the HURS Clinical Analysis Service.

The extraction, storage and confidentiality of the samples will be carried out in accordance with the regulations on the use and storage of biological samples contained in Title V of Law 14/2007, of 3 July, on Biomedical Research and Organic Law 15/1999, of 13 December, on the Protection of Personal Data.

# 7. SAFETY ASSESSMENT / ADVERSE EVENTS

## *7.1 Safety assessment*

The investigator is responsible for the detection and documentation of adverse events (AEs) throughout the trial.

It is the investigator's responsibility to report all AEs in the DCN, both observed by the investigator and spontaneously reported by the subjects, regardless of their relationship to the investigational product.

All AEs should be reported during all phases of the trial and should be followed up until resolution or until an adequate explanation is found, even if the patient has discontinued study treatment. In addition, reports of AEs occurring during the trial, including an assessment of causality, severity, and intensity, should be made on a regular basis.

Patients will be informed of the potential adverse reactions to the investigational product through the patient information leaflet and of their obligation to report any adverse events they experience. They will be provided with a means of contacting the investigators for this purpose. At all study visits, patients will be asked about the occurrence of new AEs or the evolution of pre-existing AEs.

## *7.2 Definition of adverse event*

An AE is any harmful occurrence that affects the health of a patient or trial subject being treated with a medicine, even if it is not necessarily related to that treatment.

Thus, an AE may be any unfavorable and unintended sign (including an abnormal laboratory finding), symptom, or condition (new or worsening of a pre-existing condition) that is temporally related to the use of an investigational product. For marketed medicines, it also includes failure to achieve the expected benefit (i.e. lack of efficacy), abuse and misuse.

AEs include:

- Worsening or significant or unexpected exacerbation of a pre-existing chronic or intermittent condition that is related to the condition or indication being studied.

- Any new disease discovered or diagnosed after the investigational product has been administered, even if it was present before the start of the trial.

- Signs, symptoms, or clinical consequences of a suspected interaction.

- Signs, symptoms, or clinical consequences of suspected overdose of the investigational product or concomitant medication.

- Lack of efficacy, defined as a significant failure to achieve the expected pharmacological or biological effect.

AEs may encompass events occurring before or after treatment as a result of procedures specified in the protocol (for example, modification of the subject's previous treatment or tests).

Pregnancies occurring during the trial will also be considered AEs and will be followed up.

## *7.3 Definition of Serious Adverse Events*

A serious adverse event (SAE) is any experience that suggests a significant risk, contraindication, adverse reaction or precautionary measure.

It is an SAE that, at any dose, meets at least one of the following criteria

- Causes death (death is a consequence, not an event; therefore, death cannot be used to identify the adverse event).

- Is life-threatening (the term 'life-threatening' refers to an event that, at the time it occurred, put the patient at immediate risk of death, not an event that hypothetically could have caused death if it had been more serious).

- Requires hospitalization or prolongation of an ongoing hospitalization (generally, 'hospitalization' means that the person has been admitted to hospital or emergency care for at least one night, is under observation, or is receiving treatment that could not have been provided in a physician's office or on an outpatient basis). Complications occurring during hospitalization are AEs if they prolong hospitalization or meet other severity criteria, in which case the event is classified as an SAE. Hospitalization for elective treatment of a pre-existing condition that has not worsened since baseline is not considered an AE).

- Causes permanent or significant disability/incapacity (this definition does not include experiences that may be considered minor in medical terms, such as headaches, nausea, vomiting, diarrhea, flu, or accidental injuries (e.g., sprained ankles) that may interfere with or prevent the development of daily life functions, but do not constitute a substantial change).

- Causes a congenital anomaly or birth defect.

For the purpose of reporting, suspected adverse events that are considered medically significant, even if they do not meet the above criteria, or that require intervention to prevent any of the above consequences, as well as those that result in the transmission of an infectious agent through the drug, will also be considered serious.

Pregnancies that occur during the trial are also considered serious adverse events (SAEs).

## *7.4 Definition of Adverse Reaction*

An adverse reaction (AR) is any harmful and unintended response to an investigational drug, regardless of the dose administered.

Unlike an AE, in the case of an AR, there is a suspicion of a causal relationship between the investigational drug and the adverse event.

## *7.5 Definition of Serious and Unexpected Adverse Reactions*

An unexpected adverse reaction (UAR) is any adverse reaction the nature, severity, or outcome of which is not consistent with the product information (e.g., Investigator's Brochure for an investigational product not approved for marketing, or Summary of Product Characteristics for an approved product).

The unexpected nature of an adverse reaction is based on the fact that it has not been previously observed and is not based on what would be expected based on the pharmacological properties of the product.

The concept of a serious and unexpected adverse reaction (SUAR) has been described above and in Section 7.3 of the protocol.

## *7.6 Analytical anomalies and other abnormalities*

Laboratory test results are recorded in the DCN.

Analytical abnormalities or other abnormal evaluations (e.g. vital signs) that are considered clinically significant at the investigator's discretion, that are detected during the trial or that are present at visit 0 or baseline, and that worsen significantly after the start of the trial, will be recorded as AEs (serious or non-serious, as defined). However, laboratory abnormalities or other clinically significant abnormalities related to the condition being studied will not be reported as AEs, unless the investigator considers them to be more severe than expected for the subject's condition, or unless they are present or detected at baseline and do not worsen.

If unexplained abnormal laboratory values that are clinically significant are observed, the tests will be repeated and follow-up will continue until the values return to the normal range, return to baseline values, and/or a clear explanation for the abnormality is found.

## *7.7 Assessment of intensity*

The intensity of all AEs will be graded according to the National Cancer Institute (NCI-CTCAE) version 4.0 criteria on a five-point scale (grades 1-5) and detailed in the DCN.

The determination of the possible relationship with the study treatment shall be made according to the following definitions:

| CTC Grade | Equivalent to: | Definition |
| --- | --- | --- |
| Grade 1 | Mild | Asymptomatic or only causes mild symptoms without interruption of normal daily activity (NDA); only clinical or diagnostic observations are indicated. No intervention is warranted. |
| Grade 2 | Moderate | General discomfort sufficient to limit basic daily activities appropriate for the age*. A minimal, local, or non-invasive intervention is indicated. |
| Grade 3 | Severe | Severe or medically significant, but not immediately life-threatening; debilitating; limits activities of daily living related to personal care**. Hospitalisation or extension of hospitalisation is indicated. |
| Grade 4 | Life-threatening/disabling | Life-threatening consequences. Urgent intervention is indicated. |
| Grade 5 | Death | Death related to AE. |

* Basic Activities of Daily Living include preparing meals, shopping for food and clothing, using the telephone, managing money, etc.

** Personal Care Activities of Daily Living refer to bathing, dressing and undressing, eating without assistance from another person, using the toilet, taking medication and not being confined to bed.

An AE described as “severe" should not be confused with a "serious” AE. Intensity is a category used to qualify the severity of an adverse event, and both non-serious AEs and serious AEs can be classified as intense or severe. An adverse event is defined as serious when it results in one of the predefined outcomes described in Section 7.3 of the protocol.

## *7.8 Assessment of causality*

The investigator will assess the causal relationship between the AE and the study medication.

If there is a reasonable suspicion that the study drug has a causal relationship with the adverse event, i.e. there are facts (indicators) or arguments that suggest such a relationship, the causality algorithm used by the Spanish Pharmacovigilance System (modified by Karch and Lasagna) will be considered, which uses 5 criteria:

- Temporal Sequence (TS), consistent with the administration of the drug.

- Prior knowledge (PK). Follows a known pattern of response to the suspected drug.

- Evolution after withdrawal (EW). Disappears or decreases when treatment is discontinued, or the dose is reduced.

- Re-exposure effect (RE). Occurs when the drug is reintroduced.

- Alternative cause (AC). Natural history of underlying disease, concomitant treatments or other risk factors.

The total imputability score classifies the causal relationship into 5 categories:

- UNLIKELY....................≤ 0

- CONDITIONAL...........................1-3

- POSSIBLE ....................................4-5

- PROBABLE.................................6-7

- DEFINITE..................................≥ 8

The causal relationship is DEFINITE if the AE is related to the administration of the drug, improves on discontinuation and recurs on readministration, and cannot be explained by other causes; PROBABLE if it is related to the administration of the drug, improves on discontinuation and cannot be explained by other causes; POSSIBLE, if it is related to the use of the drug but can be explained by other causes; CONDITIONAL, if it is causally related to the use of the drug but does not coincide with the AEs of the drug and can be explained by alternative causes. Finally, UNLIKELY or no causal relationship (not related) if there is no temporal sequence, does not coincide with the AEs described for the drug and can be explained by other causes.

## *7.9 Collection and Follow-up of Adverse Events*

The principal investigator will collect all AEs that occur from the moment the patient signs the informed consent until the last follow-up visit, including those caused by procedures related to the trial.

All AEs will be recorded in the patient's medical history and in the DCN.

Patients may report AEs spontaneously or they may be identified through open-ended questions, examinations, or assessments during interviews conducted at study visits. To avoid reporting biases, patients should not be asked about the specific manifestation of one or more AEs.

After the initial collection of AE data, the investigator must actively monitor the progress of each patient and provide updated information regarding their condition. In this regard, all AEs will be followed up until resolution, stabilization of the problem, an alternative explanation is found, or until it becomes impossible to continue following the subject.

At the end of the patient's participation in the study, only the following AEs will be monitored:

- Present SAE and SUAR.

- Non-serious AEs related to the investigational drug or trial procedures that persist until resolved or until an alternative explanation is found or it becomes impossible to follow up with the subject.

Once resolved, the appropriate AE page in the DCN will be updated. The investigator will ensure that the follow-up includes as many additional investigations as necessary to clarify the nature and causality of the AE. This may include additional laboratory tests or analyses, histopathology, or consultation with other healthcare professionals.

## *7.10 Notification of Serious Adverse Events*

All SAEs that occur during the study, regardless of the treatment group in which they arise, must be reported to the sponsor within one business day (24 hours) from the moment the investigator becomes aware of the event.

The notification form must always be completed as thoroughly as possible, with all specific available information, and must be signed by the investigator. The minimum information in the initial notification includes: patient identification, identification of the adverse event, date of onset, reason for considering it serious, causal relationship with the study medication, name of the person originally reporting the event, and all specific available information regarding the event. If the investigator does not have all the information about the SAE, they will not wait to receive it before reporting the event. Additional information should be reported on the same form within 24 hours of its receipt.

The preferred method for the sponsor to receive this information is by sending a fax with the notification form:

**Pharmacovigilance Unit of FIBICO**

**Fax: 957 763571**

In exceptional circumstances, and in the absence of a fax machine, notification via email or phone is accepted, with a copy of the form sent by urgent mail (or attached to the email message). The initial notification by email or phone does not replace the requirement for the investigator to complete, sign, and submit the notification form within the previously indicated deadlines.

The sponsor will review the received form and, if necessary, request additional information from the investigator.

If the investigator receives additional information regarding the AE, or if it is resolved or unlikely to change, a follow-up report must be completed and also sent via fax to the Pharmacovigilance Unit of FIBICO within 24 hours of becoming aware of it. All cases of pregnancy are considered as AEs and must therefore be reported according to the procedure described above.

The immediate notification of the SAE on the form is in addition to the collection of this data in the patient's medical history and in the DCN.

The sponsor may delegate tasks to third parties, but ultimate responsibility remains with the sponsor.

The sponsor will not actively seek adverse reactions attributable to the trial medication or procedures that occur after the subject's participation in the trial has ended. However, if the investigator reports them, the sponsor will collect and process them appropriately. Such cases will be subject to expedited reporting as required by the regulatory authority.

The sponsor must promptly report any information that may change the risk-benefit balance of the investigational product, or may require changes in the protocol or conduct of the trial:

- A qualitative change or an increase in the proportion of expected AEs that is considered clinically significant.

- SAEs that occur after the completion of a clinical trial and are reported by the investigator to the sponsor.

- New events related to the conduct of the trial or the development of the investigational product that are likely to affect the safety of subjects, such as

- AEs that may be related to the trial procedures and may change the conduct of the trial.

- A significant risk to subjects, such as the lack of efficacy of an investigational product used to treat a life-threatening condition.

- New significant safety findings from new animal studies (e.g. carcinogenicity).

- Any premature termination or temporary halt of a clinical trial of the same investigational product for safety reasons conducted in another country by the same sponsor.

- SUARs related only to a MNI, which are considered relevant because they are not subject to the general rules for accelerated reporting of individual SUARs.

- Any recommendations made by the Data Monitoring Committee that are relevant to the safety of trial subjects.

This relevant information must be reported as soon as possible and no later than 15 days after the promoter becomes aware of it. In addition, if additional relevant information becomes available, it must be reported as soon as possible.

The sponsor must communicate to all investigators involved any information that may affect the safety of trial subjects as soon as possible. They will also be informed throughout the trial of any safety issue that affects the conduct of the trial or the development of the product, including the interruption of the development program or any safety-related protocol amendments.

The trial will comply with all local regulatory requirements. In addition, the trial will comply with all requirements of the ICH Guideline for Clinical Safety Data Management, Definitions and Standards for Expedited Reporting, Topic E2A.

## *7.11 Expedited notification of SUARs to health authorities / CREC.*

The sponsor, through the vigilance Unit of FIBICO, is responsible for notifying the AEMPS, the involved CRECs, and the autonomous communities where the trial is conducted, of all SUARs collected in the study, following the procedure indicated in the current legislation.

For SUARs suspicions occurring in Spanish territory, only the official Spanish language of the State will be accepted, and the SUAR notification form (Annex xx) will be used.

The maximum notification period for an individual case of suspected SUAR will be 15 calendar days from the moment the promoter becomes aware of it. When the suspicion of SUAR has caused the patient's death, or endangered their life, the promoter will send the information within 7 calendar days from the moment they become aware of it. He will complete this information, if possible, within the following 8 days.

This information should include an assessment of the significance and implications of the findings, including relevant prior experience with the same or similar medication.

The notification can be made by fax (+34 918225076), by postal mail, or it can be delivered in person to the AEMPS (Las Mercedes Business Park Building 8, C/ Campezo 1 - 28022 Madrid) addressed to the Clinical Trials Area of the General Subdirectorate of Human Use Medicines. Suspected adverse reactions will be accompanied by a cover letter.

The AEMPS will make public the date from which it will accept electronic notifications sent by the promoters. Likewise, the AEMPS will determine the deadline from which it will no longer accept notifications in digital or paper format, except for justified reasons. Until then, the promoters will be able to continue making notifications in paper format, without prejudice to the fact that in the case of SUAR notifications occurring outside of Spain, when the SUAR is notified to Eudravigilance, additional notification to the AEMPS is not necessary.

Each of the CRECs involved in a clinical trial must be notified of all SUARs that occurred in the subjects participating at the centers within their area of influence. Likewise, the competent authority of each Autonomous Community where the trial is conducted must be notified of any suspected SUAR occurrences in the healthcare centers of their Community. In both cases, the SUAR notification form will be used for this purpose.

The promoter will annually send the researchers the information on the SUAR in an aggregated list along with a brief analysis of the provided data.

## *7.12 Annual Safety Reports.*

The annual safety reports that will include the SUARs and SAEs collected in the study since the patient is randomized will be sent by FIBICO to the AEMPS (Clinical Trials Area of the General Subdirectorate of Human Use Medicines), the Autonomous Communities, and the CRECs, within the deadlines established by current legislation.

## *7.13 Management of adverse events.*

The management of an AE is at the discretion of the investigator and is based on current good clinical practice guidelines. Any medication administered to treat an AE will be recorded in the subject's DCN.

## *7.14 Pregnancy.*

According to ICH Guideline M3, precautions should be taken to minimise the risk to the foetus or embryo when potentially fertile women are enrolled in clinical trials. These precautions include serum pregnancy testing at baseline to exclude pregnancy, the use of highly effective contraception, and continued monitoring if pregnancy occurs.

If a pregnancy occurs during the patient's participation in the trial, it will be considered an SAE and the investigator will record the pregnancy information in the CRD. In addition, the patient will be followed up to determine the outcome of the pregnancy (including abortion).

Pregnancy complications and voluntary abortions for medical reasons, as well as spontaneous abortions, should also be reported in the SAE. The investigator will promptly inform the sponsor of any SAE related to pregnancy or the newborn/infant that the investigator believes may be related to the investigational drug during the trial and after the patient has completed the trial.

Any pregnancy that occurs during the study and its outcome must be recorded and monitored to rule out abnormalities or congenital malformations. Information will be collected on:

- Normal birth, spontaneous or therapeutic abortion (any congenital anomaly detected in the aborted fetus must be documented), stillbirth, congenital anomaly.

- Neonatal death occurring within 30 days of birth.

- Death of an infant after 30 days if suspected by the investigator to be related to intrauterine exposure to the study drug.

All infants born after fetal exposure should be followed for the first 12 months after birth.

## *7.15 Deaths.*

Deaths occurring during treatment with the investigational product or within 12 weeks after discontinuation of treatment, whether or not considered to be related to treatment, must be reported. All deaths considered to be related to the trial drug at any time should be reported as SAEs, regardless of the time elapsed since the administration of the last dose of the trial product.

# 8. STATISTICAL METHODOLOGY

## *8.1 Data sets for analysis*

### 8.1.1 Intention to treat (ITT) population

The ITT population is defined as all patients who were randomly assigned, regardless of whether they received the intervention. Patients are classified according to the treatment group to which they were randomly assigned, regardless of the treatment they actually received. This population is the basis for the primary efficacy analyses.

### 8.1.2 Per protocol population (PP)

The PP dataset consists of all patients from the ITT dataset with no major protocol deviations. This is the group of patients who entered the study as planned.

Protocol deviations are defined as:

- Non-compliance with inclusion and exclusion criteria;

- Use of prohibited medications;

- Non-compliance with visit dates;

- failure to meet the primary endpoint without premature discontinuation;

- Non-compliance with the study design;

- any other deviation during the course of the trial.

### 8.1.3 Safety data set

The safety data set includes all patients enrolled in the trial who received at least one dose of any trial product.

## *8.2 Sample size determination*

The sample size calculation for the clinical trial is based on the regional disease control rate (LC rate %) at 36 months post-intervention (primary endpoint). We have assumed that the proportion of patients in the treatment arm with local disease control (LC rate %) at 36 months will be 82% (with a peritoneal recurrence rate of 18% at 36 months) and that the proportion of patients in the control arm with local disease control (LC rate %) at 36 months will be 64% (with a peritoneal recurrence rate of 36% at 36 months).

With this estimate (alpha error = 0.05, power = 0.80, two-tailed), the calculated sample size is 190 patients, with 95 patients in each arm. Taking into account an approximate loss of 5% of patients, the final N is 200 patients.

## *8.3 Types of analysis*

- Descriptive analysis for quantitative variables by calculating arithmetic means (m) and standard deviations (SD); and for qualitative variables by calculating counts, percentages and proportions (%).

- Goodness of fit to a normal distribution (normal data) is determined using the Shapiro-Wilk test. Homogeneity of variance is also checked using Levene's test.

- Comparison of means of quantitative variables between the two groups using Student's t-test (parametric test) or Mann-Whitney U test (non-parametric test).

- Comparison of proportions between groups using chi-squared tests for contingency tables; in the case of 2 x 2 tables, the chi-squared statistic with Yates' correction is used, and when any expected frequency is ≤ 5, Fisher's exact test is used.

- Associations between quantitative variables will be assessed by calculating Pearson's linear correlation coefficient (parametric test) or Spearman's correlation (non-parametric test), as appropriate.

- Survival curves will be examined using the Kaplan-Meier method and compared using the log-rank test to analyze the effect of different factors on survival.

All hypothesis contrasts will be two-sided. In all statistical tests, "significant" values are those with a 95% confidence level (p<0.05).

# 9. ADMINISTRATIVE OBLIGATIONS

## *9.1 Source documents*

Source documents are any documents, original records, electronic or paper, from which the patient's CRD data are derived.

## *9.2 Data collection and management*

The principal investigator must maintain a list of qualified individuals to whom study tasks have been delegated. All individuals authorized to make entries and/or corrections in the DCN are listed on the delegation of authority form signed by the sponsor.

Investigators must complete a DCN for each patient enrolled in the trial, collecting all data required for the trial, including laboratory data.

All forms must be completed clearly and legibly. Corrections should be made by striking out the incorrect entry (without erasing or making it illegible) and inserting the correct information, the date and the initials of the investigator or authorized representative making the correction next to the corrected part. The investigators will ensure the accuracy, integrity, legibility, and timeliness of the data in the DCN and any requested records.

When the patient has completed the trial, the principal investigator will sign the DCN and submit it to the sponsor (or designated technical staff) for filing. This also applies to DCNs of subjects who do not complete the trial.

All data required for analysis and communication will be entered into a validated database. The database will be closed once the quality control procedures for data management have been completed.

The monitor will verify compliance with the protocol and the integrity, consistency, and accuracy of the data entered into the DCN, and will ensure that the trial is conducted in accordance with Good Clinical Practice (GCP) guidelines and all applicable regulatory requirements.

Adequate and accurate records will be maintained to ensure that the conduct of the trial is fully documented, and that subsequent verification of the data is possible.

## *9.3 Investigator's file/document retention*

The investigator must maintain adequate and accurate records to ensure that the trial is fully documented and that the trial data can be subsequently verified. These documents should be divided into two distinct groups: the investigator study file and the original patient clinical records.

The investigator study file includes the protocol and amendments, ethics committee and competent authority approvals and correspondence, the template for the informed consent form, medication records, team CVs, authorization forms, and other relevant documents and correspondence.

The original clinical records of the patients include the patient's hospital records, physician and nursing notes, diary, original laboratory reports, signed informed consent forms, consultation reports, and records of patient selection and enrolment.

The investigator must retain both categories of documents described above for at least 15 years from the date the trial is completed or terminated, or for a longer period if required by local law. After this period, the documents may be destroyed in accordance with local law.

If the investigator wishes to transfer the trial documents to another person or to another location, the investigator must notify the sponsor in advance.

If the investigator cannot meet the requirements for storing some or all of the trial documents at the trial site, the investigator and the sponsor must agree on special measures to store these documents in a sealed box outside the trial site, so that they can be returned to the investigator in the event of a regulatory audit. If original documents are required for the ongoing care of the patient, appropriate copies must be obtained for storage outside the trial centre.

The investigator must provide the sponsor, upon request, with the basic data required from the trial documents or clinical records. This is particularly important if there is a suspicion of data transcription errors. Access to the full trial documentation is also necessary in the event of specific problems and/or regulatory inquiries, or if an audit is required, provided that patient confidentiality is protected.

## *9.4 Data Quality Assurance / Audits and Inspections*

To ensure compliance with good clinical practice and all applicable regulatory requirements, the sponsor may conduct a quality assurance audit. Regulatory authorities may also conduct an inspection of this trial. Audits and inspections may occur at any time during or after the trial. In the event of an audit or inspection, the investigator and the trial site will agree to allow the auditor or inspector direct access to all relevant records and documents of the trial (provided that the confidentiality of trial subjects is protected) and to provide the necessary time, both their own and that of their staff, to discuss the findings and other pertinent matters with the auditor or inspector.

The investigator/institution must facilitate access to the original data/documents for trial monitoring, audits, ethics committee reviews, and regulatory inspections. In accordance with all applicable regulations, the investigator and the trial site must allow authorised representatives of the sponsor, regulatory authority(ies), and ethics committee direct access to the original medical records of patients for verification of trial-related procedures and data.

This direct access includes the examination, analysis, review, and reproduction of any record or report that is relevant to the evaluation of the trial. The investigator must inform the subject and obtain the subject's consent to allow designated representatives access to the subject's study-related records without breaching the subject's confidentiality. Verification of DCN data must be by direct inspection of the original documents.

## *9.5 Publication policy*

The results of this trial may be published or presented at scientific meetings.

The conditions for publication will be in accordance with the provisions of Royal Decree 223/2004, of 6 February, which regulates Clinical Trials of Medicinal Products, Article 38, which states that:

1. The sponsor is obliged to publish the results, both positive and negative, of authorised clinical trials in scientific journals.

2. The funding received by the author for the conduct of the trial and the sources of funding shall be disclosed.

3. The anonymity of trial subjects is maintained at all times.

4. The results or conclusions of the clinical trials will preferably be reported in scientific publications before being made available to the non-healthcare public. Procedures whose efficacy has not yet been determined will not be prematurely, sensationally, or exaggeratedly publicized.

Once the study has been completed and the statistical report has been produced, the research team will write the final study report, which will be submitted to the relevant Ethics Committee (CREC), the Spanish Agency for Medicines and Medical Devices (AEMPS) and any health authorities that request it. This final report will be used as the basis for preparing manuscripts for publication in medical journals.

## *9.6 Study monitoring*

The monitoring of the trial will be conducted in accordance with the recommendations of ICH Topic E6. Guideline for Good Clinical Practice. CPMP/ICH/135/95.

The monitor will contact the site prior to subject enrolment to review the protocol and study procedures with the site staff. In addition, the monitor will be responsible for inspecting the case report forms (CRFs) and other relevant data (ensuring that subject confidentiality is maintained in accordance with regulatory requirements) at regular intervals throughout the trial to verify compliance with the protocol and the integrity, consistency, and accuracy of the data entered. The extent, nature, and frequency of site visits will depend on the objectives and/or endpoints of the trial, the purpose of the trial, the complexity of the design, and the recruitment rate.

During these contacts, the monitor will:

- Review the progress of the clinical trial and the pace of patient recruitment.

- Review the data collected during the clinical trial.

- Review the original documents.

- Identify any problems and discuss their resolution.

This is done to verify that:

- The data are authentic, accurate, and complete.

- The safety and rights of trial subjects are protected.

- The trial is conducted in accordance with the current protocol (and any amendments), good clinical practice guidelines, and all applicable regulatory requirements.

The monitor has access to the patient's medical records at the investigator's request. The investigator agrees to provide the monitor with direct access to all relevant documents and to devote the necessary time, both personally and with his team, to discuss findings and other relevant matters with the monitor.

The investigator must allow the monitor, the sponsor's internal auditors, and representatives of the regulatory authorities to inspect all trial-related documents and relevant medical or hospital records for confirmation of DCN data.

The monitor's obligations are described as follows:

1. Work in accordance with the sponsor's standard operating procedures (SOPs).

2. Visit the investigator before, during, and after the trial to ensure compliance with the protocol.

3. Ensure that data are recorded accurately and completely.

4. Confirm that informed consent has been obtained from all subjects prior to their inclusion in the study.

5. Ensure that the investigators and the site where the research is being conducted are suitable for the purpose.

6. Ensure that both the principal investigator and his or her staff have been adequately informed and that prompt communication between the investigator and the monitor is maintained at all times.

7. Ensure that the storage, distribution, and documentation of investigational drugs are safe and adequate.

8. Submit monitoring visit reports and all relevant contacts with the investigator to the sponsor.

# 10. REGULATORY AND ETHICAL OBLIGATIONS

## *10.1 Regulations*

This clinical trial will be conducted in accordance with the protocol, the tenets of the current revised version of the Declaration of Helsinki (Seoul, 2008) (Appendix 3), and the applicable regulatory requirements, in particular the ICH Tripartite Guideline "Good Clinical Practice", Royal Decree 223/2004 regulating clinical trials with medicinal products in Spain, and the EU Clinical Trials Directive (2001/20/EC) concerning the provisions of the Member States on the application of good clinical practice in the conduct of clinical trials of medicinal products for human use.

The investigator is aware, upon signing the protocol, that he/she is bound by the instructions and procedures described therein and will ensure that the established provisions are strictly followed.

The sponsor will obtain approval from the health authorities (AEMPS) in accordance with all applicable legal requirements specific to the country.

The trial will not commence until the AEMPS approval and the CREC approval have been obtained.

The principal investigator is responsible for ensuring that this protocol, the centre's informed consent document, and any other information presented or provided to potential subjects (e.g., advertisements or information to support or supplement informed consent) are reviewed and approved by the relevant CRECs. The investigator agrees to provide the CREC with direct access to all relevant documents. The CREC must be constituted in accordance with all applicable regulatory requirements.

## *10.2 Informed consent*

Informed consent must be obtained from the patient prior to enrolment in the trial. The content and procedure for obtaining consent must comply with all applicable regulatory requirements.

The investigator is responsible for obtaining written informed consent from each patient participating in the study, after explaining in an understandable manner the nature, objectives, methods, expected benefits, and possible risks of the study.

The investigator must also explain to patients that they are completely free to refuse to participate in the trial or to withdraw from the trial at any time for any reason.

For patients who are incompetent or unable to give legal consent, written consent must be obtained from their authorized legal representative. If the patient and their legal representative are unable to read, an impartial witness must be present throughout the informed consent process. After the patient and/or their legal representative have given their verbal consent to participate in the study, the witness will sign the document to confirm that the information contained in the informed consent form has been explained and fully understood.

The subject will give consent by signing the appropriate form. For this purpose, each form must bear the signature of both the investigator and the patient.

The DCN for this trial includes a section to document the patient's informed consent, which must be properly completed.

If new safety information becomes available that significantly changes the risk/benefit assessment, the DCN will be reviewed and updated as necessary. All patients, including those already receiving treatment, must be informed of the new information and given a copy of the updated consent form to re-consent to continue in the trial.

## *10.3 Confidentiality*

All information related to the trial is considered confidential.

To ensure the confidentiality of the trial data, access will be restricted to the principal investigator and his/her team of collaborators, the designated technical staff, the monitor, the sponsor, the ethics committee, and the relevant health authorities.

The contents of the data collection notebooks, the documents generated during the trial, and the database will be protected from unauthorized use by persons not involved in the research, and will therefore be treated as strictly confidential and will not be disclosed to third parties, except as specified in the previous sections.

The sponsor guarantees that the data will be processed in accordance with the security measures established in Organic Law 15/1999 on the Protection of Personal Data. By signing the informed consent form, the participant agrees to this use of the study data. This consent has no expiry date. The participant may revoke it at any time, but must do so in writing.

All materials, information (oral or written), and unpublished documents provided to the investigators, including this protocol, the data collection sheets, and the investigator's manual, must be considered the property of the sponsor.

It is the investigator's responsibility to maintain and ensure the confidentiality of all documents and results generated in the course of the trial, except those that are required by law to be disclosed. The data and/or materials from the trial may not be disclosed, in whole or in part, by the investigator or the investigator's staff to any unauthorized person without the prior formal written consent of the sponsor.

The investigator will ensure that the anonymity of the subjects is maintained and that their identities are protected from unauthorized third parties. To maintain the confidentiality of patient data and to protect the doctor-patient relationship, each participating patient will be assigned a unique reference number. In the CRDs or other documents submitted to the sponsor, the names of the subjects should not appear, and the identification code should be used instead. The investigator must keep a record of subject recruitment showing the codes and names.

All data and information generated by the trial site in the course of the trial (with the exception of the subjects' medical records) will be kept confidential by the investigator and the rest of the trial site staff. This information and data will not be used by the investigator or other site staff for any purpose other than the conduct of the trial. These restrictions do not apply to:

- Information that remains in the public domain through no fault of the researcher or the center’s staff.

- Information that must be disclosed confidentially to an Ethical Review Committee (ERC) for the sole purpose of evaluating the clinical trial.

- Information that must be disclosed in order to provide appropriate medical care to a subject of the trial.

- Results of the trial that may be published as described in the following paragraph.

## *10.4 Good Clinical Practice (GCP) Responsibilities*

### 10.4.1 Sponsor's Responsibilities

The sponsor's responsibilities are as follows:

1. Establish Standard Operating Procedures (SOPs).

2. Sign the protocol and any amendments with the investigator.

3. Select the most appropriate person to conduct the trial and ensure that this person performs this function as specified in the protocol.

4. Provide all available basic and clinical information about the investigational product and update it throughout the trial.

5. Request reports from the CRECs and authorisation from the General Directorate of Pharmacy and Health Products and, if necessary, inform them or obtain their authorisation, without prejudice to communication with the Autonomous Communities in the event of amendments, protocol violations or study suspensions.

6. Use a pharmaceutical laboratory to supply the drugs to be tested, ensuring that good manufacturing practice (GMP) is followed and that samples are properly packaged and labelled.

7. He is also responsible for ensuring that there is a proper procedure for the handling, storage and use of these drugs at the centre where the trial is being conducted.

8. Appoint the monitor who will oversee the progress of the trial.

9. Notify the health authorities and ethics committees involved in the trial:

a. Any serious and unexpected adverse reactions that may be related to the investigational treatments, occurring inside or outside Spain.

b. Any information from animal studies suggesting a significant risk to humans, including any evidence of teratogenicity or carcinogenicity. In such cases, the sponsor, together with the investigator, will take the necessary measures to protect the trial subjects.

10. Provide the investigator and, when appropriate, the ethics committees with all relevant information to which they have access during the trial.

11. Provide financial compensation to subjects in the event of injury or death related to the trial.

12. Agree with the investigator on obligations regarding data processing, reporting, and publication of results. In any case, the sponsor is responsible for preparing final or interim reports and submitting them to the General Directorate of Pharmaceuticals and Health Products.

### 10.4.2 Responsibilities of the investigator

The investigator's responsibilities are:

1. Agree and sign the project protocol with the sponsor.

2. To have a thorough understanding of the properties and characteristics of the investigational product(s) to be used.

3. Obtain informed consent from patients before enrolling them in the trial.

4. Accurately collect, record and report data.

5. To notify the sponsor immediately of any serious or unexpected adverse events.

6. To ensure that all persons involved in the trial respect the confidentiality of all information relating to the trial subjects.

Failure of an investigator to comply with the protocol, SOPs, good clinical practice (GCP), and/or relevant regulatory requirements must result in immediate intervention by the sponsor to ensure compliance.

If monitoring and/or auditing reveals serious and/or persistent noncompliance by an investigator, the sponsor must remove the investigator from the trial. If an investigator is removed because of noncompliance, the sponsor must immediately notify the regulatory authorities.

## *10.5 Insurance*

In accordance with current legislation, and in particular Royal Decree 223/04, the promoter shall take out an insurance policy covering the liabilities of the promoter, the principal investigator and their collaborators, and the hospital or centre where the clinical trial is being carried out, against any damage or harm that may affect the patient's health during the trial and in the year following its completion.

## *10.6 Funding*

## *10.7 Conditions for amending the protocol*

In order to ensure the conditions of the trial and the validity of the statistical analysis of the data, neither the investigator nor the sponsor may modify the agreed and stipulated conditions of the trial as described in this protocol.

Any amendment must be in writing, stating the reasons, and signed by all parties involved. The amendment will then become an integral part of the protocol. If amendments require the approval of ethics committees and/or authorities, such approval must be obtained. Any amendment that alters the risk-benefit balance for the patient must, once signed by the investigator, be submitted to the AEMPS and CREC for evaluation and approval.

Protocol amendments must be prepared by the investigator. The investigator may not modify the protocol himself.

Any changes to the protocol, the informed consent document, or any other information that the CREC has approved for distribution to potential subjects must be submitted to the same CREC for its information and approval, and to the health authorities (if required), in accordance with the regulatory requirements. The investigator is responsible for ensuring that the CREC reviews and, if necessary, approves these amended documents, and such approval must be obtained before any changes can be made (except, for example, changes necessary to eliminate an immediate risk to trial subjects). If an amendment to the informed consent form is made, the investigator must comply with all applicable regulatory requirements for its use, including obtaining CREC approval of the amended document before new subjects consent to participate in the trial using that version of the form.

## *10.8 Conditions for termination of the trial*

If the trial is terminated or suspended, the sponsor must promptly notify the investigator and the regulatory authorities of the termination or suspension and the reason for the termination or suspension. The sponsor or the investigator should promptly inform the CREC, stating the reason for the termination or suspension, as required by the applicable regulatory requirement(s).

Both the sponsor and the investigator reserve the right to terminate their participation in the trial under the circumstances agreed in the contract with the centre. If this becomes necessary, both parties will individually decide on the procedures to be followed after review and consultation. Upon termination of the trial, the sponsor and the investigator will ensure that due consideration is given to the protection of the patients' interests.

**11. BIBLIOGRAPHY**

1. J. Ferlay, D.M. Parkin, E. Steliarova-Foucher. Estimates of cancer incidence and mortality in Europe in 2008. Eur J Cancer 2010;46(4):765–81

2. Snaebjornsson P., Coupe V.M.H., Jonasson L. pT4 stage II and II colon cáncer carry the worst prognosis in a nationwide survival análisis. Shepherd’s local peritoneal involvement revisited. Int. J. Cancer 2013. 00, 00-00.)

3. Shepherd NA, Baxter KJ, Love SB. The prognostic importance of peritoneal involvement in colonic cáncer: a prospective evaluation. Gastroenterology 1997; 112:1096-102).

4. Hompes D, Tiek J, Wolthuis A. HIPEC in T4a colon cáncer: a defendable traeatment to improve the oncologic outcome? Annals of Oncology 2012, 23; 3123-3129)

5. Sugarbaker PH. Peritoneal carcinomatosis drugs and diseases. Boston : Kluwer; 1996

6. Verwaal VJ, van Ruth, deBree E.Randomized trial of cytoreduction and hyperthermic intraperitoneal chemotherapy vs systemic chemotherapy and palliative surgery in patients peritoneal cacinomatosis of colorectal cáncer. J Clin Oncol2003; 21:3737-3743.

7. Elias D, Lefevre JH, Chevalier J. Complete cytoreductive surgery plus intraperitoneal chemohyperthermia with oxaliplatin for peritoneal carcinomatosis of colorectal origin. J Clin Oncol. 2009; 27:681-685.

8. Cao C, Yan TD, Black D, Morris DL. A systematic review and meta-analysis of cytoreductive surgery with perioperative intraperitoneal chemotherapy for peritoneal carcinomatosis of colorectal origin. Ann Surg Oncol. 2009; 16: 2152-2162

9. Elias D, Goere D, Di Pietrantonio D. Results of systematic second-look surgery in patients with high risk of developing colorectal peritoneal carcinomatosis. Ann Surg. 2008; 247:445-450

10. Yan TD, Black D, Sugarbaker PH. A systematic review and meta-analysis of the randomized controlled trial son adjuvant intraperitoneal chemotherapy for resectable gastric cáncer. Ann Surg Oncol. 2007; 14: 2702-2713

11. Tentes AAK, Spiliotis ID, Korakianitis OS. Adjuvant perioperative intraperitoneal chemotherapy in locally advanced colorectal carcinoma: preliminary results. ISRN Surgery volumen 2011, article ID 529876.

12. Sammartino P, Simone S, Biachi D. Preventin of peritoneal metastasis from colon cancer in high risk patients: preliminary results of surgery plaus prophylactic HIPEC. Gastroenterology Research and practice. 2011. ID: 141585.

13. Baratti D, Kasamura S, Deraco M. Colorectal cancer peritoneal metastasis: second look laparotomy, prophilactic HIPEC or both? Ann Surg, 2014. May 30.

14 . Noura S, Ohue M, Shingai T. Effects of intraperitoneal chemotherapy with mitomycin c on the prevention of peritoneal recurrence in colorectal cáncer patients with positive peritoneal lavage cytology findings. Ann Surg Oncol. 2011; 18: 396-404

15. Rufián Peña S, Muñoz Casares F.C., Briceño Delgado F.J. Radical surgery-peritonectomy and intraoperative intraperitoneal chemotherapy for the treatment of peritoneal carcinomatosis in recurrent or primary ovarian cancer. Journal of Surgical Oncology (2006); 94; 316-324.

16. A.Arjona-Sánchez, F.C. Muñoz-Casares, S. Rufián-Peña. Pseudomyxoma peritonei treated by cytoreductive surgery and hyperthermic intraperitoneal chemotherapy: results from a single centre. Clin Transl Oncol 2011, 13 (4): 261-267.

17. A. Arjona-Sánchez, F.C Muñoz Casares, A.Casado Adam. Outcome of patients with aggressive pseudomyxoma peritonei treated by cytoreductive surgery and intraperitoneal chemotherapy. World J Surg 2013, 2013 Jun; 37(6):1263-70.

18. F.C. Muñoz-Casares, S. Rufián, A.Arjona-Sánchez. Neoadjuvant intraperitoneal chemotherapy with paclitaxel for the radical surgical treatment of peritoneal carcinomatosis in ovarian cáncer: a prospective pilot study. Cancer Chemotherapy and Pharmacology. 2011. Jul; 68(1):267-74.

# 12. ANNEXES

**Annex 1. Subject information and informed consent sheet**

**INFORMED CONSENT - PATIENT INFORMATION**

Before signing this informed consent form, please read the following information carefully and ask any questions you may have..

**TITLE OF THE PROJECT: “**Multicentre, randomised study to evaluate the adjuvant effect of hyperthermic intraperitoneal chemotherapy (HIPEC) with mitomycin C associated with surgery versus surgery alone in locally advanced colorectal carcinoma.”

**Nature:**

For the treatment of the tumour disease he suffers from, he needs an intervention that includes several techniques to achieve the elimination of the tumour.

This study aims to analyse the effect of intraperitoneal intraoperative hyperthermic intraperitoneal chemotherapy (HIPEC) on the possibility of locoregional tumour recurrence.

This intervention is performed to treat tumour disease with the intention of eliminating all tumour remnants, whenever possible, and to complete the elimination of visible (macroscopic) tumour tissue with the eradication of non-visible or microscopic tumour tissue by hyperthermic intraperitoneal chemotherapy (HIPEC).

Surgical treatment consists of removal of all infiltrated regions or regions suspected of tumour infiltration + resection of target organs (organs where locoregional recurrence is most frequent) such as the greater omentum, ileocaecal appendix, hepatic round ligament and, in the case of women and postmenopausal women, resections of both ovaries will be included. After surgical excision and in the same operative act, the administration of HIPEC will be carried out or not, after which the appropriate anastomosis or derivative ostomies will be carried out if the case requires it. If hyperthermic intraperitoneal chemotherapy (HIPEC) is administered, it will last for 60 minutes and will be carried out with the drug mitomycin C using a continuous infusion system.

The doctor has advised me that the procedure requires the administration of anaesthesia, and that it is possible that during or after the procedure blood and/or blood products may be necessary, of which I will be informed by the anaesthesia department of the risks involved.

**Relevance:**

The procedure is intended to eliminate the possibility of locoregional recurrence of the disease you are suffering from, since, even if the tumor is removed and you subsequently receive complementary chemotherapy treatment, the risk of recurrence at the locoregional level is high.

This procedure of radical surgery with or without intraperitoneal intraoperative chemotherapy is a novel treatment that combines the two treatment modalities (surgery + chemotherapy). To know that, the application of intraperitoneal chemotherapy is a common technique in our Unit for patients who present a more advanced neoplastic disease than the one you present, obtaining a significant benefit in both disease-free survival and overall survival. The combination of surgery + intraoperative chemotherapy in your case could improve results in terms of local control of the disease with minimal associated toxicity.

It is planned to include a total of 200 patients, who will be divided into two groups of 100 patients each. One experimental group will receive intraperitoneal chemotherapy while the other control group will not. Membership in one group or the other will be randomized, i.e., randomly. You have a 50% chance of being in one group or the other.

**Implications for patients:**

- - - - Participation in this trial is completely voluntary.
      - The patient may withdraw from the study at any time, without explanation, and without this having any repercussions on his or her medical care.
      - All personal data obtained in this study are confidential and will be treated in accordance with the Spanish Personal Data Protection Act 15/99.
      - The donation/information obtained will be used exclusively for the specific purposes of this study.

**Risks of research for the patient:**

The complications associated with the application of intraperitoneal chemotherapy, different from the complications inherent to surgery, usually resolve well with medical treatment (drugs, serum, etc.). However, they may sometimes require a reoperation, generally an emergency one. It is exceptional, but not impossible, that these problems could lead to death.

The most frequent:

- Wound infection

- Bleeding of the surgical wound

- Acute retention of urine, hematuria (blood in urine) requiring the administration of saline solution.

- Phlebitis (inflammation of the vein walls).

- Prolonged pain in the area of the operation.

- Delayed recovery of intestinal transit.

The most severe:

- - - - If intestinal sutures are performed, fistulas (abnormal communications) may occur due to failure of the sutures to heal.
      - Intra-abdominal hemorrhage
      - Intra-abdominal infection.
      - Abdominal collections
      - Intestinal obstruction.
      - Laparotomy dehiscence (opening of the abdominal wound).
      - Hematologic toxicity due to chemotherapy.
      - Reintervention and sometimes death

*Risks associated with the intraperitoneal administration of mitomycin C for chemotherapy with or without hyperthermia::*

The most common toxicity described during adjuvant intraperitoneal administration has been abdominal pain, being mild on most occasions and very rarely of severe intensity. Following abdominal pain, other gastrointestinal toxicities observed were nausea, vomiting, indigestion, anorexia, and stomatitis, which were related to the cytostatic dose. Diarrhea and constipation have been described with no clear relationship to dosing.

Some patients have experienced hypersensitivity symptoms such as facial flushing, pruritus, which subsided with antihistamine medication.

Asthenia and fatigue have been reported as dose-dependent.

Peripheral neuropathy has also been reported in some studies.

So far, no significant cardiac toxicity or alopecia have been observed with intraperitoneal therapy.

In some patients hepatic or renal toxicity was observed, which were not severe, and only required dose reduction in subsequent cycles.

Myelosuppression is another related adverse effect, with leukopenia, neutropenia (sometimes associated with fever) and thrombocytopenia also being dose-dependent.

Your doctor can discuss with you the possible existence of other less common effects. There may also be other adverse effects not known at this time. However, due to the short intraperitoneal exposure time when administered intraoperatively, all these adverse effects in relation to the cytostatic are minimized.

-In relation to intraperitoneal hyperthermia, the most outstanding side effects have been hemodynamic alterations due to vasodilatation and/or tachycardia, although there may be other less frequent or related to the cytostatic used.

If you require additional information you can contact our Oncologic Surgery Unit staff at the following telephone number: 957010439 or e-mail: 957010439.: [cirughrs.hrs.sspa @juntadeandalucia.es](mailto:cirughrs.hrs.sspa@juntadeandalucia.es)

**INFORMED CONSENT - WRITTEN CONSENT OF THE PATIENT**

**“**Randomized, multicenter study to evaluate the adjuvant effect of hyperthermic intraperitoneal chemotherapy (HIPEC) with mitomycin C associated with surgery versus surgery alone in locally advanced colorectal carcinoma.”.

I (First and Last Name):

.............................................................................................................................

- I have read the information document accompanying this consent (Patient Information).

- I have been able to ask questions about the study “Multicenter, randomized study to evaluate the adjuvant effect of hyperthermia intraperitoneal chemotherapy (HIPEC) with mitomycin C associated with surgery versus surgery alone in locally advanced colorectal carcinoma”.

- I have received sufficient information about the study. I have spoken with the reporting health care professional:

…………………………………………………………

- I understand that my participation is voluntary and I am free to participate or not in the study.

- I have been informed that all data obtained in this study will be kept confidential and will be treated in accordance with the Organic Law of Personal Data Protection 15/99.

will be treated in accordance with the Organic Law on Personal Data Protection 15/99.

- I have been informed that the donation/information obtained will only be used for the specific purposes of the study.

specific purposes of the study.

- I wish to be informed of my genetic and other personal data obtained in the course of the research, including unexpected discoveries that may occur, provided that this information is necessary to avoid serious harm to my health or that of my biological relatives.

Si / No

I understand that I can withdraw from the study:

- Whenever I want

- Without having to explain myself

- Without repercussion on my medical care.

I freely give my agreement to participate in the project entitled: “Multicenter, randomized study to evaluate the adjuvant effect of hyperthermia intraperitoneal chemotherapy (HIPEC) with mitomycin C associated with surgery versus surgery alone in locally advanced colorectal carcinoma”.

Firma del paciente (o representante legal en su caso) Firma del profesional sanitario informador

First and last name:………………. First and last name: ………..

Date: ……………………………… Date: ……………………….

## *Annex 2. Example of study label.*

Promotor: FIBICO (Fundación para la Investigación Biomédica de Córdoba)

Inv. Principal: Dr.

Código Protocolo: Nº EudraCT:

Centro:

Código Paciente: ______________

Medicamento en investigación:

Forma farmacéutica:

Vía de administración:

Dosis:

Nº lote:

Condiciones de conservación: No conservar a temperatura superior a 25 °C.

Fecha de caducidad: _____/_______/_____ (dd/mm/aa)

Fecha dispensación: _____/_______/_____ (dd/mm/aa)

**EXCLUSIVAMENTE PARA USO EN ENSAYO CLÍNICO**

## *Annex 3. Serious Unexpected Adverse Reaction Reporting Occurring in Spain.*

| NOTIFICACION DE SOSPECHA DE  REACCION ADVERSA PARA  MEDICAMENTOS EN INVESTIGACIÓN | CODIGO DE PROTOCOLO (promotor)……………….  Nº EUDRACT/ Nº Protocolo AEMPS……………….. | Nº NOTIFICACION (Promotor) |
| --- | --- | --- |
| Notificación realizada a Eudravigilance 🞎 SI 🞎 NO | PACIENTE Nº | Nº NOTIFICACION |

| 1a. PAÍS | 2. FECHA DE NACIMIENTO | | | 2a. EDAD | 3. SEXO | 3a. PESO | 3b. TALLA | | 4-6. FECHA DE INICIO DE LA REACCIÓN | | |
| --- | --- | --- | --- | --- | --- | --- | --- | --- | --- | --- | --- |
|  | DÍA | MES | AÑO |  | 🞎 HOMBRE  🞎 MUJER |  |  | | DÍA | MES | AÑO |
| 7. DESCRIPCIÓN DE LA REACCIÓN ADVERSA (Incluyendo resultados relevantes de exploración o de laboratorio, y la fecha de finalización, si procede). | | | | | | | | 8-13b. CRITERIOS DE GRAVEDAD/ DESENLACE  🞎 FALLECIMIENTO  🞎 LA VIDA DEL PACIENTE HA ESTADO EN PELIGRO  🞎 HOSPITALIZACIÓN  🞎 PROLONGACIÓN HOSPITALIZACIÓN  🞎 INCAPACIDAD PERMANENTE O SIGNIFICATIVA  🞎 RA CLINICAMENTE RELEVANTE  Desenlace  🞎 PERSISTENCIA DE LA RA  🞎 RECUPERACIÓN SIN SECUELAS  🞎 RECUPERACIÓN CON SECUELAS  🞎 DESCONOCIDO | | | |

II. INFORMATION ON THE INVESTIGATIONAL DRUG

| 14. MEDICAMENTO SOSPECHOSO | 15. DOSIS  DIARIA | 16. VÍA | 17. ENFERMEDAD EN ESTUDIO | | 18. FECHAS DE  INICIO FINAL | | 19. DURACIÓN DEL  TRATAMIENTO |
| --- | --- | --- | --- | --- | --- | --- | --- |
|  |  |  |  | |  |  |  |
| 20. ¿REMITIÓ LA REACCIÓN AL SUSPENDER LA  MEDICACIÓN?  🞎 SI 🞎 NO 🞎 NO PROCEDE | | 20a. ¿REMITIÓ LA REACCIÓN AL REDUCIR LA  DOSIS?  🞎 SI 🞎 NO 🞎 NO PROCEDE | | 21. ¿REAPARECIÓ LA REACCIÓN AL ADMINISTRAR DE NUEVO LA MEDICACIÓN?  🞎 SI 🞎 NO 🞎 NO PROCEDE | | | |

III. CONCOMITANT MEDICATIONS AND MEDICAL HISTORY

| 22. MEDICAMENTOS CONCOMITANTES (Márquese con un asterisco el o los medicamentos sospechosos) | 22a. DOSIS  DIARIA | 22b. VÍA | 22c. FECHAS DE  INICIO FINAL | | 22d. MOTIVO DE LA PRESCRIPCIÓN |
| --- | --- | --- | --- | --- | --- |
|  |  |  |  |  |  |
|  |  |  |  |  |  |
|  |  |  |  |  |  |
|  |  |  |  |  |  |
| 23. DATOS IMPORTANTES DE LA HISTORIA CLÍNICA (ej. diagnósticos, alergias, embarazos, etc.) | | | | | |

IV. PROMOTER AND RESEARCHER INFORMATION

| 24a. NOMBRE Y DIRECCION DEL PROMOTOR | | 24b. NOMBRE Y DIRECCION DEL INVESTIGADOR |
| --- | --- | --- |
| 24c. CODIGO DE LABORATORIO  (Nº AEMPS) | 25a. TIPO DE INFORME  INICIAL  SEGUIMIENTO | 24c. TECNICO DEL PROMOTOR QUE INFORMA  NOMBRE:  TELEFONO:  FIRMA: |
| 24e. FECHA DEL INFORME | 24f. FECHA DE ENTRADA AEM | 25b. SE ADJUNTA INFORME COMPLEMENTARIO |

**GENERAL RULES**

1. This form is to be used only for reporting suspected unexpected serious adverse reactions (ARs) occurring with investigational drugs.

2. Suspected fatal or life-threatening AR (those which, without immediate therapeutic intervention, would have resulted in the death of the patient) will be reported within a maximum of 7 calendar days; if all the information is not available, it may be completed within an additional 8 days. Other suspicions of serious and unexpected AR will be reported within a maximum of 15 days.

3. When the available space is insufficient, an additional information sheet shall be added, correctly identified with the name of the promoter and the number assigned to the notification. This information may include the causality assessment made by the reporting technician.

**SPECIFIC INSTRUCTIONS**

1. The protocol code is the code assigned by the sponsor to identify the trial. The sponsor's notification number is the one used by the sponsor for archiving. In the case of follow-up information, the same number should be used or, if changed, the number of the initial report should be used. The shaded “Notification No.” space should be left blank.

2. The age should be given in years, months, weeks or days as appropriate, but always indicating it. If the age is not known precisely, at least the age group to which it belongs should be stated (e.g., infant, child, adolescent, adult, elderly).

7. The AR shall be described in full, indicating the date of its completion and including the results of the complementary examinations or laboratory tests considered to be of interest. This notification may be accompanied by as many reports as deemed appropriate for the proper interpretation of the clinical picture suspected of being an adverse reaction.

8-13. The categories are not mutually exclusive. Attendance in an Emergency Department of a Hospital for less than 24 hours shall not be considered hospitalization.

14. Investigational medicinal products shall be identified, if possible, by their generic name (DOE or INN), indicating the trade name when available, or alternatively, by the proposed name or laboratory code for the product.

15. In case the administration is not daily, try to describe it with one of the following possibilities: cyclic, weekly, monthly, yearly or number of times it has been used (in this case, put the dose of each intake, not the total).

17. The pathological process of the patient for whom the investigational product is intended, or “healthy volunteer” in the case of such a patient, shall be stated.

19. The duration of treatment until the onset of the adverse reaction shall be recorded.

22. It will be explicitly indicated if no concomitant drugs have been taken. If any of the concomitant drugs are considered suspicious, they should be marked with an asterisk (e.g. * AMOXICILLIN). Drugs used to treat the adverse reaction will be excluded..

## *Anexo 4. World Medical Association Declaration of Helsinki*

**HELSINKI DECLARATION OF THE WORLD MEDICAL ASSOCIATION**

**WORLD MEDICAL ASSOCIATION**

Ethical Principles for Medical Research Involving Human Subjects Adopted by the 18th World Medical Assembly, Helsinki, Finland, June 1964 and amended by the 29th World Medical Assembly, Tokyo, Japan, October 1975 35th World Medical Assembly, Venice, Italy, October 1983 41st World Medical Assembly, Hong Kong, September 1989 48th General AssemblySomerset West, South Africa, October 1996 52nd General Assembly, Edinburgh, Scotland, October 2000

Note of Clarification of Paragraph 29, added by the WMA General Assembly, Washington, D.C. 2002

Note of Clarification of Paragraph 30, added by the WMA General Assembly, Tokyo 2004 59th General Assembly, Seoul, Korea, October 2008

*A. INTRODUCTION*

1. The World Medical Association (WMA) has promulgated the Declaration of Helsinki as a set of proposed ethical principles for medical research involving human subjects, including research on identifiable human material and information.

The Declaration should be considered as a whole and one paragraph should not be applied without consideration of all other relevant paragraphs.

2. Although the Statement is primarily intended for physicians, the WMA urges other participants in medical research involving human subjects to adopt these

Principles.

3. The physician's duty is to promote and safeguard the health of patients, including those involved in medical research. The physician's knowledge and conscience must be subordinated to the fulfillment of that duty.

4. The Declaration of Geneva of the World Medical Association binds the physician to the formula “the health of my patient first and foremost”, and the International Code of Medical Ethics states that: “A physician shall consider the best interests of the patient when providing medical care”.

5. Progress in medicine is based on research that ultimately must include human studies. Populations that are underrepresented in medical research must have appropriate access to participation in research.

6. In medical research involving human subjects, the welfare of the research subject must always take precedence over all other interests.

7. The primary purpose of medical research involving human subjects is to understand the causes, course, and effects of disease and to improve preventive, diagnostic, and therapeutic interventions (methods, procedures, and treatments). Even the best current interventions must be continually evaluated through research to ensure that they are safe, effective, efficient, accessible and of high quality.

8. In the practice of medicine and medical research, most interventions involve some risks and costs.

9. Medical research is subject to ethical standards that serve to promote respect for all human beings and to protect their health and individual rights. Some research populations are particularly vulnerable and in need of special protection. These include those who cannot give or refuse consent on their own and those who may be vulnerable to coercion or undue influence.

10. Physicians should consider the ethical, legal and juridical norms and standards for research involving human subjects in their own countries, as well as current international norms and standards. No national or international ethical, legal or regulatory requirement should be permitted to diminish or eliminate any of the protections for research subjects set forth in this Declaration.

*B. PRINCIPLES FOR ALL MEDICAL RESEARCH*

11. In medical research, it is the physician's duty to protect the life, health, dignity, integrity, right to self-determination, privacy and confidentiality of the personal information of research subjects.

12. Medical research involving human subjects should conform to generally accepted scientific principles and should be supported by a thorough knowledge of the scientific literature and other relevant sources of information, as well as by properly conducted laboratory and animal experiments, when appropriate. Care should also be taken to ensure the welfare of the animals used in the experiments.

13. When conducting medical research, adequate attention should be paid to factors that may damage the environment.

14. The design and method of any study involving human subjects should be clearly described in a research protocol. The protocol should always make reference to the ethical considerations involved and should indicate how the principles set forth in this Declaration have been considered. The protocol should include information on funding, sponsors, institutional affiliations, following possible conflicts of interest and incentives for study subjects, and provisions for dealing with or compensating subjects who have suffered harm as a result of their participation in the research. The protocol should describe arrangements for post-trial access to interventions identified as beneficial in the study or access to other appropriate care or benefits.

15. The research protocol should be submitted for consideration, comment, advice, and approval to a research ethics committee prior to the start of the trial. This committee should be independent of the investigator, the sponsor, or any other undue influence.

The committee should consider the laws and regulations in force in the country where the research is being conducted, as well as current international standards, but these should not be allowed to diminish or eliminate any of the protections for research subjects set forth in this Statement. The committee has the right to monitor ongoing trials. The investigator has the obligation to provide monitoring information to the committee, especially about any serious adverse events. No changes in the protocol should be made without the consideration and approval of the committee.

16. Medical research involving human subjects should be conducted only by persons with appropriate scientific training and qualifications. Research on healthy patients or volunteers requires the supervision of a physician or other appropriately qualified and competent health care professional. Responsibility for the protection of research subjects should always rest with a physician or other health care professional and never with the research participants, even if they have given their consent.

17. Medical research in a disadvantaged or vulnerable population or community is justified only if the research responds to the health needs and priorities of this population or community and if there is a reasonable possibility that the population or community on which the research is conducted will benefit from its results.

18. Every medical research project involving human subjects should be preceded by a careful comparison of the risks and costs to the persons and communities participating in the research, compared to the foreseeable benefits to them and to other persons or communities affected by the disease under investigation.

19. Every clinical trial should be enrolled in a publicly available database before the first person is accepted.

20. Physicians should not participate in human research studies unless they are confident that the inherent risks have been adequately assessed and can be satisfactorily addressed. They should immediately discontinue the ongoing experiment if they observe that the risks involved outweigh the expected benefits or if there is conclusive evidence of positive or beneficial results.

21. Medical research involving human subjects should only be conducted when the importance of its purpose outweighs the inherent risk and costs to the research subject.

22. The participation of competent individuals in medical research must be voluntary. While it may be appropriate to consult family members or community leaders, no competent individual should be included in a study unless they freely consent.

23. All necessary precautions must be taken to safeguard the privacy of the individual participating in the research and the confidentiality of their personal information, as well as to minimize the impact of the research on their physical, mental, and social integrity.

24. In medical research involving competent human subjects, each potential individual must receive adequate information regarding the objectives, methods, sources of funding, possible conflicts of interest, institutional affiliations of the researcher, calculated benefits, foreseeable risks, and discomforts arising from the experiment, as well as any other pertinent aspects of the research. The potential subject must be informed of their right to participate or not in the research and to withdraw their consent at any time without facing repercussions. Special attention should be given to the specific information needs of each potential individual, as well as to the methods used to deliver the information. After ensuring that the individual has understood the information, the physician or another appropriately qualified person should then request, preferably in writing, the informed and voluntary consent of the individual. If consent cannot be obtained in writing, the process for achieving it must be documented and formally witnessed.

25. For medical research involving identifiable human material or data, the physician must normally obtain consent for the collection, analysis, storage and re-use. There may be situations where it is impossible or impractical to obtain consent for such research, or where it could compromise the validity of the research. In this situation, the research can only be conducted after it has been reviewed and approved by a research ethics committee.

26. In obtaining informed consent to participate in research, the physician must exercise special care when the potential subject is in a dependent relationship with the physician or when consent is given under pressure. In such a situation, informed consent must be sought from a suitably qualified person who has no connection with that relationship..

27. If the potential individual is incapacitated, the physician must obtain informed consent from the legal representative. Such persons should not be included in research that has no potential to benefit them, unless the research is designed to promote the health of the population represented by the potential individual and cannot be conducted on competent persons, and the research involves only minimal risk and cost.

28. If a potential research subject who is considered incompetent is able to give his or her consent to participate or not to participate in the research, the physician must obtain that consent in addition to the consent of the legal representative. The potential subject's refusal must be respected..

29. Research involving persons who are physically or mentally incapable of giving consent, such as unconscious patients, may be conducted only if the physical/mental condition preventing informed consent is a necessary characteristic of the population being studied. In these circumstances, the physician must obtain informed consent from a legal representative. If such a representative is not available and the research cannot be delayed, the study may proceed without informed consent, provided that the specific reasons for enrolling persons with a condition that prevents them from giving informed consent are stated in the research protocol and the study is approved by an institutional review board. Consent to remain in the research must be obtained from the individual or a legal representative as soon as possible.

30. Authors, directors and editors all have ethical obligations in relation to the publication of their research. Authors have a duty to make the results of their research involving human subjects available to the public and are responsible for the integrity and accuracy of their reports. They must adhere to ethical standards in the dissemination of information. Negative, inconclusive, and positive results should be published or otherwise made available to the public. Publication must include the source of funding, institutional affiliations, and conflicts of interest. Reports of research that do not adhere to the principles outlined in this statement should not be accepted for publication.

*C. PRINCIPLES APPLICABLE WHEN MEDICAL RESEARCH IS COMBINED WITH MEDICAL CARE*

31. The physician may combine medical research with medical care only to the extent that such research has a justified potential preventive, diagnostic or therapeutic value and if the physician has good reason to believe that participation in the study will not adversely affect the health of the patients involved in the research.

32. The potential benefits, risks, costs and effectiveness of any new intervention must be assessed by comparing it with the best existing proven intervention, except in the following circumstances:

- The use of a placebo or no treatment is acceptable in trials for which there is no existing proven intervention.

- When, for methodological, scientific, and urgent reasons, the use of a placebo is necessary to determine the efficacy and safety of an intervention that does not involve risk, serious adverse effects, or irreversible harm to patients receiving the placebo or no treatment.

Great care must be taken not to abuse this option..

33. At the end of the research, all patients participating in the study have the right to be informed of their results and to share in any benefits, such as access to interventions identified as beneficial in the study or other appropriate care or benefits.

34. The physician must fully inform the patient about the aspects of care related to the research. The patient's refusal to participate or decision to withdraw from a study should never interfere with the doctor-patient relationship.

35. When proven interventions for the care of a patient have been ineffective or do not exist, the physician, after seeking expert advice and with the informed consent of the patient or an authorized legal representative, may authorize the use of unproven interventions if, in the physician's judgment, there is some hope of saving life, restoring health, or alleviating suffering. Whenever possible, such interventions should be investigated to assess their safety and efficacy. In all cases, this new information must be recorded and, where appropriate, made available to the public.


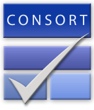
CONSORT 2010 checklist of information to include when reporting a randomised trial*

| Section/Topic | Item No | Checklist item | Reported on page No |
| --- | --- | --- | --- |
| Title and abstract | | | |
|  | 1a | Identification as a randomised trial in the title | 1 |
|  | 1b | Structured summary of trial design, methods, results, and conclusions (for specific guidance see CONSORT for abstracts) | 4 |
| Introduction | | | |
| Background and objectives | 2a | Scientific background and explanation of rationale | 5 |
|  | 2b | Specific objectives or hypotheses | 5 |
| Methods | | | |
| Trial design | 3a | Description of trial design (such as parallel, factorial) including allocation ratio | 6 |
|  | 3b | Important changes to methods after trial commencement (such as eligibility criteria), with reasons | 6 |
| Participants | 4a | Eligibility criteria for participants | 6 |
|  | 4b | Settings and locations where the data were collected | 6 |
| Interventions | 5 | The interventions for each group with sufficient details to allow replication, including how and when they were actually administered | 6 |
| Outcomes | 6a | Completely defined pre-specified primary and secondary outcome measures, including how and when they were assessed | 6 |
|  | 6b | Any changes to trial outcomes after the trial commenced, with reasons | 6 |
| Sample size | 7a | How sample size was determined |  |
|  | 7b | When applicable, explanation of any interim analyses and stopping guidelines | 6 |
| Randomisation: |  |  |  |
| Sequence generation | 8a | Method used to generate the random allocation sequence | 6 |
|  | 8b | Type of randomisation; details of any restriction (such as blocking and block size) | 6 |
| Allocation concealment mechanism | 9 | Mechanism used to implement the random allocation sequence (such as sequentially numbered containers), describing any steps taken to conceal the sequence until interventions were assigned | 6 |
| Implementation | 10 | Who generated the random allocation sequence, who enrolled participants, and who assigned participants to interventions | 6 |
| Blinding | 11a | If done, who was blinded after assignment to interventions (for example, participants, care providers, those assessing outcomes) and how | 6 |
|  | 11b | If relevant, description of the similarity of interventions |  |
| Statistical methods | 12a | Statistical methods used to compare groups for primary and secondary outcomes | 6 |
|  | 12b | Methods for additional analyses, such as subgroup analyses and adjusted analyses | 6 |
| Results | | | |
| Participant flow (a diagram is strongly recommended) | 13a | For each group, the numbers of participants who were randomly assigned, received intended treatment, and were analysed for the primary outcome | 7 |
|  | 13b | For each group, losses and exclusions after randomisation, together with reasons | 7 |
| Recruitment | 14a | Dates defining the periods of recruitment and follow-up | 7 |
|  | 14b | Why the trial ended or was stopped | 7 |
| Baseline data | 15 | A table showing baseline demographic and clinical characteristics for each group | 7 |
| Numbers analysed | 16 | For each group, number of participants (denominator) included in each analysis and whether the analysis was by original assigned groups | 7 |
| Outcomes and estimation | 17a | For each primary and secondary outcome, results for each group, and the estimated effect size and its precision (such as 95% confidence interval) | 7 |
|  | 17b | For binary outcomes, presentation of both absolute and relative effect sizes is recommended | 7 |
| Ancillary analyses | 18 | Results of any other analyses performed, including subgroup analyses and adjusted analyses, distinguishing pre-specified from exploratory |  |
| Harms | 19 | All important harms or unintended effects in each group (for specific guidance see CONSORT for harms) |  |
| Discussion | | | |
| Limitations | 20 | Trial limitations, addressing sources of potential bias, imprecision, and, if relevant, multiplicity of analyses | 8 |
| Generalisability | 21 | Generalisability (external validity, applicability) of the trial findings | 8 |
| Interpretation | 22 | Interpretation consistent with results, balancing benefits and harms, and considering other relevant evidence | 8 |
| Other information | | |  |
| Registration | 23 | Registration number and name of trial registry | 3 |
| Protocol | 24 | Where the full trial protocol can be accessed, if available | Suppl |
| Funding | 25 | Sources of funding and other support (such as supply of drugs), role of funders | 3 |

*We strongly recommend reading this statement in conjunction with the CONSORT 2010 Explanation and Elaboration for important clarifications on all the items. If relevant, we also recommend reading CONSORT extensions for cluster randomised trials, non-inferiority and equivalence trials, non-pharmacological treatments, herbal interventions, and pragmatic trials. Additional extensions are forthcoming: for those and for up to date references relevant to this checklist, see [www.consort-statement.org](http://www.consort-statement.org).
